# Enzymatic bypass of an *N*^6^-deoxyadenosine DNA–ethylene dibromide–peptide cross-link by translesion DNA polymerases

**DOI:** 10.1016/j.jbc.2021.100444

**Published:** 2021-02-20

**Authors:** Pratibha P. Ghodke, Gabriela Gonzalez-Vasquez, Hui Wang, Kevin M. Johnson, Carl A. Sedgeman, F. Peter Guengerich

**Affiliations:** Department of Biochemistry, Vanderbilt University School of Medicine, Nashville, Tennessee, USA

**Keywords:** DNA polymerase, DNA damage, DNA cross-ink, DNA cross-link repair, DNA alkylation, DNA replication, DNA–protein interaction, AGT, *O*^6^-alkylguanine DNA-alkyl transferase (MGMT), dha, dehydroalanine, DMSO, dimethylsulfoxide, EDB, ethylene dibromide, ESI, electrospray ionization, HR, homologous recombination, MALDI, matrix-assisted laser desorption ionization (mass spectrometry), MS, mass spectrometry, MSH, *O*-(mesitylsulfonyl)hydroxylamine, NER, nucleotide excision repair, Pol, DNA polymerase, TLS, translesion synthesis, UDG, uracil DNA glycosylase, UPLC, ultraperformance liquid chromatography

## Abstract

Unrepaired DNA–protein cross-links, due to their bulky nature, can stall replication forks and result in genome instability. Large DNA–protein cross-links can be cleaved into DNA–peptide cross-links, but the extent to which these smaller fragments disrupt normal replication is not clear. Ethylene dibromide (1,2-dibromoethane) is a known carcinogen that can cross-link the repair protein *O*^6^-alkylguanine-DNA alkyltransferase (AGT) to the N6 position of deoxyadenosine (dA) in DNA, as well as four other positions in DNA. We investigated the effect of a 15-mer peptide from the active site of AGT, cross-linked to the N6 position of dA, on DNA replication by human translesion synthesis DNA polymerases (Pols) η, ⍳, and κ. The peptide–DNA cross-link was bypassed by the three polymerases at different rates. In steady-state kinetics, the specificity constant (*k*_cat_/*K*_m_) for incorporation of the correct nucleotide opposite to the adduct decreased by 220-fold with Pol κ, tenfold with pol η, and not at all with Pol ⍳. Pol η incorporated all four nucleotides across from the lesion, with the preference dT > dC > dA > dG, while Pol ⍳ and κ only incorporated the correct nucleotide. However, LC-MS/MS analysis of the primer-template extension product revealed error-free bypass of the cross-linked 15-mer peptide by Pol η. We conclude that a bulky 15-mer peptide cross-linked to the N6 position of dA can retard polymerization and cause miscoding but that overall fidelity is not compromised because only correct pairs are extended.

The preservation of genome integrity is vital for the proper development of an organism. Cells are subjected to multiple endogenous and exogenous agents capable of causing lesions and affect multiple DNA transaction processes (*e.g.*, replication, repair, and transcription) (1). Covalent DNA–protein cross-links are bulky lesions and can be toxic if left unrepaired (2); they are formed from both exogenous and endogenous sources. Accumulation of these cross-links has been associated with aging, cancer, neurodegeneration, and Ruijs–Aalfs syndrome (2, 3, 4, 5, 6), and there has been considerable interest in both DNA–protein cross-links and proteases that can act on them (6). Several laboratories have shown that both reversible and irreversible DNA–protein cross-links can be formed under physiological conditions, that there are DNA-stimulated proteases that can act on these, and that DNA–protein cross-links can be bypassed and can miscode (2, 7, 8, 9, 10, 11, 12, 13, 14, 15, 16, 17, 18, 19). The number of cross-links in a cell has been estimated to be high (20) but the exact number is not known. There is now evidence that DNA–protein adducts may be important in disease states, *e.g.*, cross-linking was reported to be increased following ischemic reperfusion in cardiomyocytes (21). The list of cross-linked sites includes multiple DNA bases (G, C, T, A) and their modifications (*e.g.*, *N*^7^-Me G (12), abasic sites (13), and 5-formyl dC (10, 11)). The list of proteins in the cross-links includes histones (10, 12), HMCES (13), and numerous other proteins (21, 22, 23, 24, 25). Some reversible lysine cross-links (Schiff bases) can also destabilize DNA and cause cleavage (12, 26). However, the existence of a DNA–protein cross-link is not *a priori* evidence for miscoding (22, 23).

Some DNA–protein cross-links are common, and cells have specific enzymes to act on them. One example is tyrosyl-DNA phosphodiesterases (TDP), which are enzymes capable of breaking down the covalent bond between DNA and DNA topoisomerases (27). The activity of these enzymes is limited by substrate accessibility, suggesting that the adduct needs first to be hydrolyzed to a peptide (28). Recently DNA-activated proteases (6) and the proteasome have been suggested to be involved in proteolysis by cleaving large DNA–protein cross-links to DNA–peptide cross-links (18, 29, 30). Some DNA-dependent proteases bind to ubiquitin, or small ubiquitin-like modifier (SUMO), indicative of a role for posttranslational modification in proteolysis (31, 32, 33). Ubiquitination is a key step for proteolysis by the nuclear DNA-dependent metalloprotease SPRTN (18). SPRTN uses an accessory process (*i.e.*, ubiquitination) to repair cross-links because the ubiquitin controls its activity (34). SPRTN-mediated proteolysis also depends on the location of protein cross-links on the DNA strands (30). Thus, SPRTN has various limitations with respect to DNA–protein cross-link proteolysis. FAM111A is also known to act on DNA–protein cross-links and contains a trypsin-like domain (35).

Repair mechanisms can target different components of a DNA–protein cross-link (*i.e.*, the DNA molecule, the cross-link bond, or protein) and may depend on the stage of the cell cycle (27, 28, 36). Most DNA–protein cross-links are too large for DNA polymerase bypass (37), and proteases are believed to cleave the proteins to smaller peptides that can be repaired by nucleotide excision repair (NER) (38) or homologous recombination (HR) or bypassed by translesion synthesis (TLS) polymerases (39). NER and HR have been shown to remove protein cross-links smaller than 10 kDa (40). Mammalian TLS polymerases include Y-family Pol η, ⍳, and κ and Rev1 (41), which have low processivity and fidelity (39). Only in a few cases has the mutagenic potential of a DNA–peptide cross-link been analyzed (42), and essentially all of the work in this area has been done with either uncharacterized cross-links or synthetic models.

*O*^6^-Alkylguanine DNA-alkyltransferase (AGT, or MGMT), a ∼22 kDa protein, is a crucial repair system for protection from alkylating agents (43). It directly removes and transfers alkyl groups from the O6 position on guanine to the Cys-145 located in its active site. Ethylene dibromide (EDB, or 1,2-dibromoethane) is a known carcinogen that had formerly been used as a gasoline additive, soil fumigant, and pesticide (44, 45, 46, 47). Overexpression of AGT paradoxically increased EDB toxicity and the incidence of base pair mutations in bacterial and mammalian cells (48, 49). Cys-145 of AGT reacts with EDB *via* a nucleophilic substitution that results in a half-mustard intermediate, which cyclizes to form an unstable episulfonium ion that can form a covalent bond with DNA (Fig. 1). The AGT-EDB episulfonium ion can react with nucleobases at different positions, including the N7, N2, N1, and O6 atoms of dG and the N6 atom of dA (50, 51). The DNA-binding properties of AGT facilitate formation of these specific lesions. EDB is also known to cross-link DNA with the tripeptide glutathione (GSH) in a similar mechanism (52). Thus, this DNA–protein cross-link is directly relevant to a practical problem in toxicology, and other 1,2-dihaloalkanes and other bis-functional electrophiles are also relevant (53, 54).

**Figure 1:**
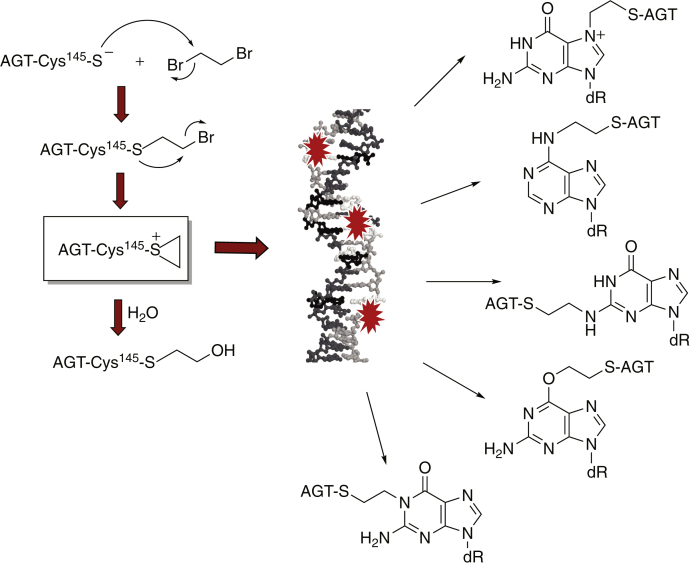
**AGT-DNA cross-links induced by ethylene dibromide** (50)**.**

We developed a procedure for the synthesis and characterization of a DNA–peptide cross-link at the N6 position of dA with a 15-mer peptide from the active site of AGT in order to investigate its effect on DNA replication by TLS polymerases. Full-length extension and single nucleotide insertion assays, steady-state kinetics, and LC-ESI-MS/MS analysis were performed to determine the efficiency and fidelity at the *N*^6^-dA-adducted peptide.

## Results

### Synthesis and characterization of a 15-mer peptide cross-linked to an oligonucleotide

**Figure 2:**
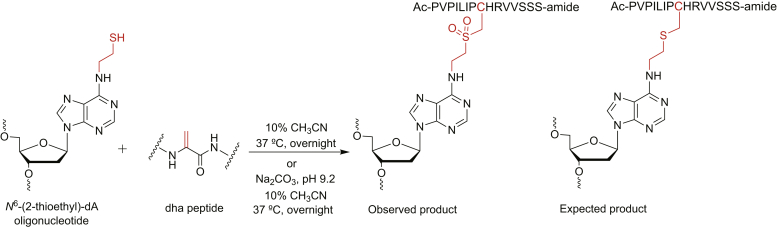
**Synthetic strategy for 15-mer peptide cross-linked at the N6 atom of dA in an oligonucleotide**.

A 15-mer peptide cross-linked to an oligonucleotide (*N*^6^-dA) was synthesized using the 15-mer peptide (acyl-PVPILIPCHRVVSSS-amide, AGT residues 138–152, with Cys-150 changed to Ser to permit exclusive modification at Cys-145) and a 6-chloropurine-containing oligonucleotide as outlined in Figure 2 (Scheme S1–S2, See Table S1 for oligonucleotide sequences). The peptide was treated with *O*-(mesitylsulfonyl)hydroxylamine (MSH), yielding amination of the cysteine to produce a dehydroalanine (dha) residue in its place (Scheme S1). The dha peptide was purified by HPLC (Fig. S1) and characterized by positive ESI-MS/MS (Figs. S2 and S3; Table S2). The 6-chloropurine-containing oligonucleotide was subjected to nucleophilic substitution with cystamine (Scheme S2), and the cystamine-containing oligonucleotide was purified by HPLC (Fig. S4) and characterized by MALDI MS (Fig. S5). Reduction of the cystamine-containing oligonucleotide with DTT yielded *N*^6^-(2-thioethyl)dA in the oligonucleotide, used for the next step without any purification (Fig. 2). Finally, the dha peptide was coupled with the thiol-containing oligonucleotide to obtain a 15-mer peptide cross-linked at the N6 position of dA *via* a two-carbon linker (Fig. 2). Alternatively, the 15-mer peptide cross-linked to DNA was also synthesized by a previously reported method (55).

**Figure 3:**
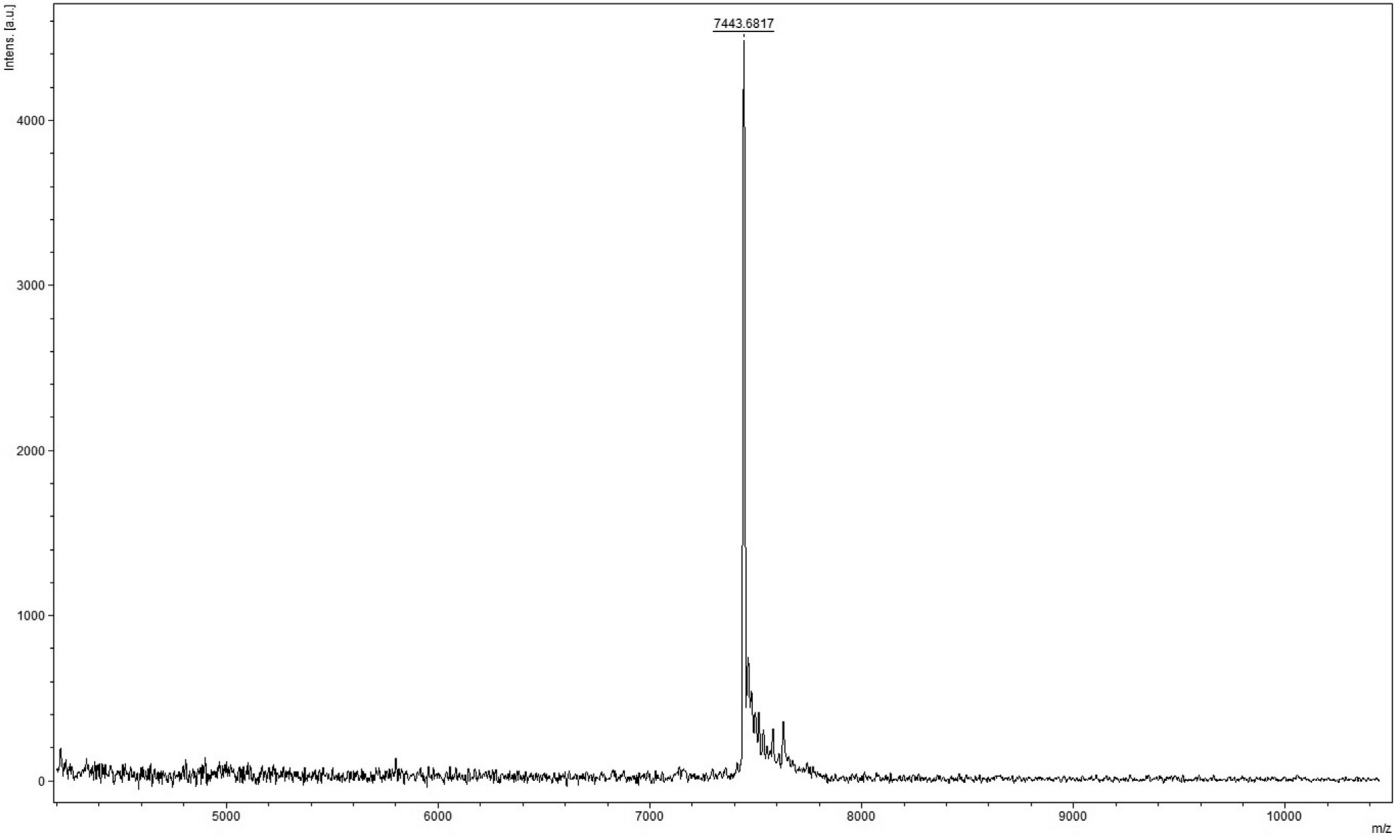
**MALDI mass spectrum of oxidized 15-mer peptide-*N***^**6**^**-dA oligonucleotide cross-link.** Expected mass [M + H]^+^ 7443.8107, observed mass [M + H]^+^ 7443.6817.

The DNA–peptide cross-link was purified by gel electrophoresis (Fig. S6) and characterized by MALDI MS as well as ESI-MS (Figs. 3 and S7). This structure is analogous to the AGT-DNA cross-link induced by EDB (50), but an oxidized sulfur-bearing adduct was observed with an additional mass of 32 a. m. u. (Figs. 2, 3, and S7) which was characterized using LC-MS/MS analysis. The DNA moiety was hydrolyzed with HF to obtain a peptide adducted with adenine (Fig. S8). In mass spectral analysis (positive mode), two major *m/z* ions were observed, *m/z* 613.66 (+3) and 919.99 (+2) (Fig. S9), indicative of an oxidized sulfur atom, which was further confirmed from the fragmentation pattern (Fig. S10 and Table S3).

### Bypass of 15-mer peptide-N^6^-dA oligonucleotide cross-link by TLS hPols η, ι, and κ

**Figure 4:**
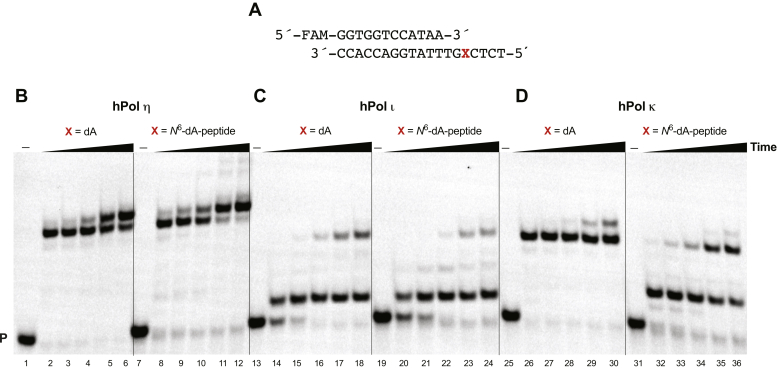
**Full-length extension by hPol η, ɩ, and κ in the presence of all four dNTPs.***A*, 12-mer primer and 19-mer template DNA sequences, where X is dA or 15-mer peptide cross-linked at *N*^6^-dA. Reactions were done in the presence of: (*B*) 20 nM hPol η; (*C*) 40 nM hPol ɩ; and (*D*) 20 nM hPol κ. All experiments were done at 37 °C for 0, 5, 10, 20, 60, and 120 min (*i.e.*, lanes, 1–6, 7–12, 13–18, 19–24, 25–30, 31–36). P, FAM-labeled 12-mer DNA primer.

Full-length extension and single nucleotide insertion assays were carried out with TLS polymerases. Full-length primer extension reactions (“running start”) were performed in the presence of all four dNTPs using a 12-mer primer (Fig. 4*A*). The *N*^6^-dA-peptide lesion in the template affected each polymerase differently. With the DNA–peptide cross-link, hPol η fully extended the primer with almost similar efficiency as the unmodified template (Fig. 4*B*, lanes 2–6 and 8–12). hPol ι mainly produced a single nucleotide incorporation product (Fig. 4*C*, lanes 20–24), similar to the unmodified template. The lesion also affected hPol κ activity, and a two-nucleotide incorporated product (stalled at lesion site) was produced along with a small amount of fully extended product (Fig. 4*D*, lanes 32–36).

**Figure 5:**
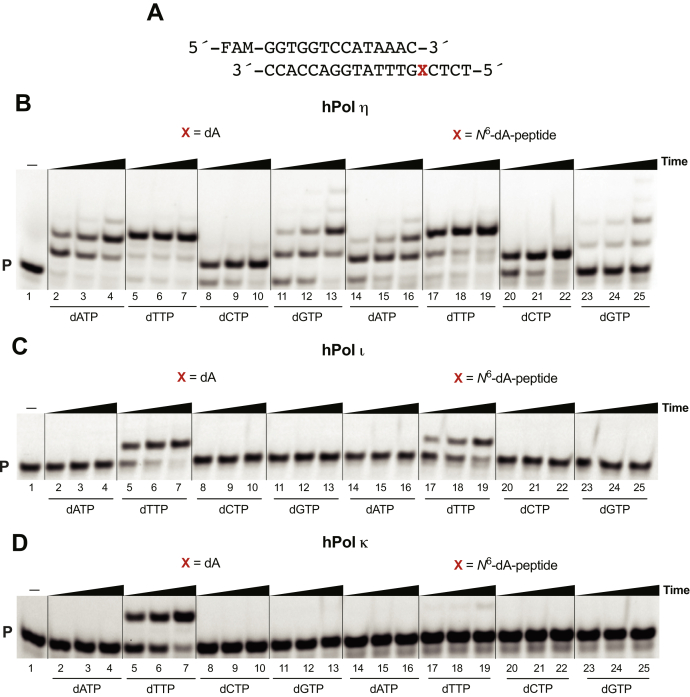
**Single nucleotide insertion by hPol η, ɩ, and κ.***A*, 14-mer primer and 19-mer template sequences, where X is dA or *N*^6^-dA-peptide. Reactions were done in the presence of: (*B*) 5 nM hPol η; (*C*) 5 nM hPol ɩ; and (*D*) 5 nM hPol κ. All experiments were done at 37 °C for 0, 5, 10, and 30 min. Lanes 2–4 and 14–16, dATP; lanes 5–7 and 17–19, dTTP; lanes 8–10 and 20–22, dCTP; lanes 11–13 and 23–25, dGTP. P, FAM-labeled 14-mer DNA primer.

Single nucleotide insertion reactions were conducted in the presence of individual dNTPs, using a 14-mer primer to assess, which is added across from the lesion (Fig. 5*A*). hPol η preferentially added dTTP across from dA and the *N*^6^-dA–peptide cross-linked template, at similar rates. The incorporation preference for the control template was dTTP > dATP > dGTP > dCTP (Fig. 5*B*, lanes 2–13) and for the *N*^6^-dA-peptide template was dTTP > dATP > dCTP > dGTP (Fig. 5*B*, lanes 14–25). hPol ι and κ only added dTTP across both templates (Fig. 5, *C* and *D*). hPol κ had the slowest rate of incorporation of dTTP opposite to the *N*^6^-dA-peptide adduct (Fig. 5*D*, lanes 17–19).

### hPol η-mediated miscoding potential of 15-mer peptide-N^6^-dA oligonucleotide cross-link

**Figure 6:**
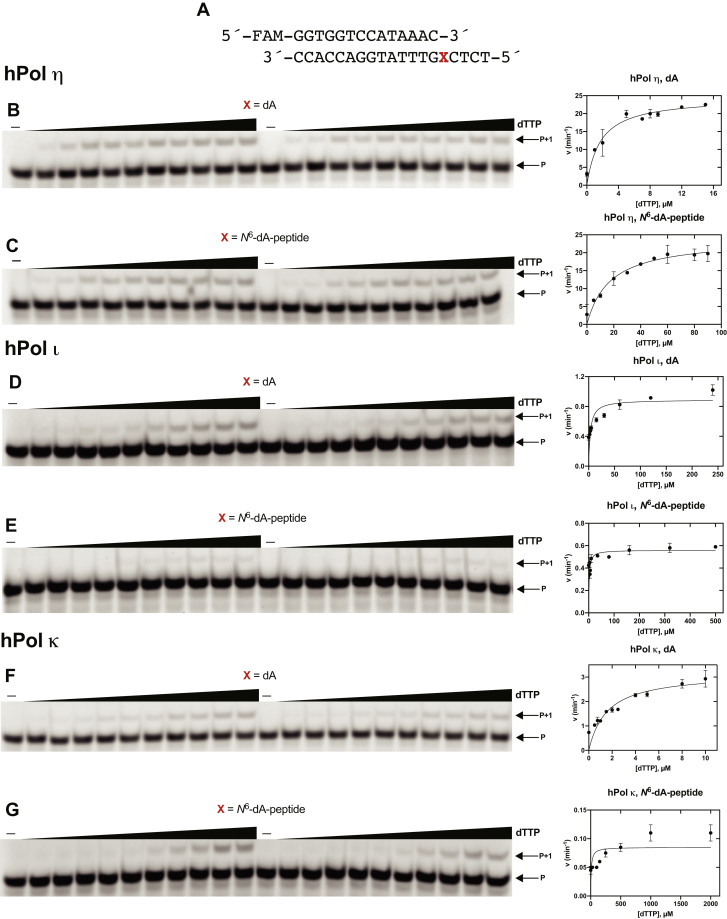
**Steady-state kinetic analysis of dTTP insertion by hPol η, ɩ, and κ.***A*, 14-mer primer and 19-mer DNA template sequences, where X is dA or *N*^6^-dA-peptide. Reactions were done at 37 °C using hPol η: (*B*) 0.09 nM and (*C*) 0.1 nM; hPol ɩ: (*D*) 0.01 and (*E*) 2 nM; hPol κ: (*F*) 2 nM and (*G*) 2.5 nM. Varying concentrations of dTTP were used: (*B*) 0–15 μM; (*C*) 0–90 μM; (*D*) 0–240 μM; (*E*) 0–500 μM; (*F*) 0–10 μM; (*G*) 0–2000 μM. Reactions were carried out for: (*F*) 8 min; (*D* and *E*) 10 min; (*B* and *C*) 12 min; (*G*) 21 min. P, FAM-labeled 14-mer DNA primer. Data points shown are means ± SD.

Steady-state kinetics were done to determine the efficiency and frequency of misincorporation with the *N*^6^-dA-adducted peptide. Reactions were done using a 14-mer primer, a single nucleotide at varying concentrations, and a particular reaction time for each polymerase (Figs. 6 and S11; Table 1). hPol η, ɩ, and κ-mediated insertion efficiency of the correct base (*i.e.*, dTTP) is shown in Figure 6, *B*–*G*. hPol η-mediated misincorporations (*i.e.*, dATP, dCTP, and dGTP) are presented in Figure S11.

**Table 1:** Steady-state kinetic analysis of insertion opposite to *N*^6^-dA-cross-linked 15-mer peptide

5′-FAM-GGTGGTCCATAAAC 3′-CCACCAGGTATTTGXCTCT-5′						
Polymerase	X	dNTP	*K*_m_, μM	*k*_cat_, min^−1^	*k*_cat_/*K*_m_, μM^−1^ min^−1^	*f*^a^
hPol η	dA	dATP	3.5 ± 1.4	1.7 ± 0.2	0.49 ± 0.15	0.035
		dTTP	1.7 ± 0.3	24.5 ± 1.2	14.1 ± 2.4	1
		dCTP	3.7 ± 0.6	1.4 ± 0.07	0.39 ± 0.05	0.027
		dGTP	14.5 ± 2.4	2.9 ± 0.13	0.21 ± 0.03	0.015
	*N*^6^-dA-peptide	dATP	8.5 ± 2.5	1.2 ± 0.1	0.140 ± 0.03	0.099
		dTTP	17 ± 3.4	24 ± 1.4	1.4 ± 0.2	1
		dCTP	3 ± 0.5	0.9 ± 0.04	0.32 ± 0.04	0.231
		dGTP	20 ± 2	1.5 ± 0.02	0.074 ± 0.006	0.053
hPol ⍳	dA	dTTP	3 ± 1	0.9 ± 0.06	0.23 ± 0.08	1
	*N*^6^-dA-peptide	dTTP	2 ± 1.2	0.5 ± 0.05	0.28 ± 0.16	1
hPol κ	dA	dTTP	1.5 ± 0.3	3.2 ± 0.23	2.03 ± 0.34	1
	*N*^6^-dA-peptide	dTTP	9.5 ± 4.8	0.09 ± 0.008	0.009 ± 0.004	1

a Misincorporation frequency (*f*) = (*k*_cat_/*K*_m_)_incorrect_/(*k*_cat_/*K*_m_)_correct_.

The specificity constant (*k*_cat_/*K*_m_) for the insertion of correct nucleotide (*i.e.*, dTTP) opposite to the adduct by hPol η was 1.4 ± 0.2 μM^−1^ min^−1^ (Table 1), tenfold lower than for insertion across the unmodified template. However, the presence of the adduct did not greatly affect the specificity constant of hPol ɩ—specificity constants of dTTP insertion across the adduct and the control template were 0.28 ± 0.16 and 0.23 ± 0.08 μM^−1^ min^−1^ (Table 1), respectively. The specificity constant for incorporation by hPol κ was 220-fold lower than the control. hPol η had the highest specificity constant for insertion of dTTP across the lesion and inserted all four nucleotides. The specificity constants for dATP, dCTP, and dGTP insertion across from the adducted peptide were decreased 3.5-, 1.2-, and 2.8-fold, compared with the unmodified template.

### hPol η-mediated primer extension analysis using LC-ESI-MS/MS

Steady-state kinetic analysis showed insertion of all dNTPs opposite to the adducted peptide by hPol η as compared with hPol ⍳ and κ (Table 1), and mass spectral analysis was performed to determine if other miscoding events occurred with hPol η (56). For full-length extension reactions, a 2′-deoxyuridine (dU)-containing 12-mer primer was used. After full-length extension, the reaction mixture was treated with uracil DNA glycosylase (UDG) and piperidine (Fig. S12) (56). Fully extended products and relative yields were calculated by LC-MS/MS analysis and are summarized in Table 2.

**Table 2:** Summary of products of extension of template–primer complexes by hPol η analyzed by LC-ESI-MS/MS

Primer: Template:		5′-FAM-GGTGGTCCA**U**AA-3′ 3′-CCACCAGGTATTTG**X**CTCT -5′		
X	Sequence	Yield (%)	Observed *m/z*	Base added
dA	5′-pAAAC**T**GAGAG-3′	50	1053.9 (−3)	T, plus blunt end addition of A and G
	5′-pAAAC**T**GAGAA-3′	50	1048.64 (−3)	
*N*^6^-dA-peptide	5′-pAAAC**T**GAGA-3′	50	944.27 (−3)	T
	5′-pAAAC**T**GAGAG-3′	28	1053.82 (−3)	T, plus blunt end addition of A and G
	5′-pAAAC**T**GAGAA-3′	22	1048.64 (−3)	

The bold and underlined T is the base inserted opposite the adduct X.

**Figure 7:**
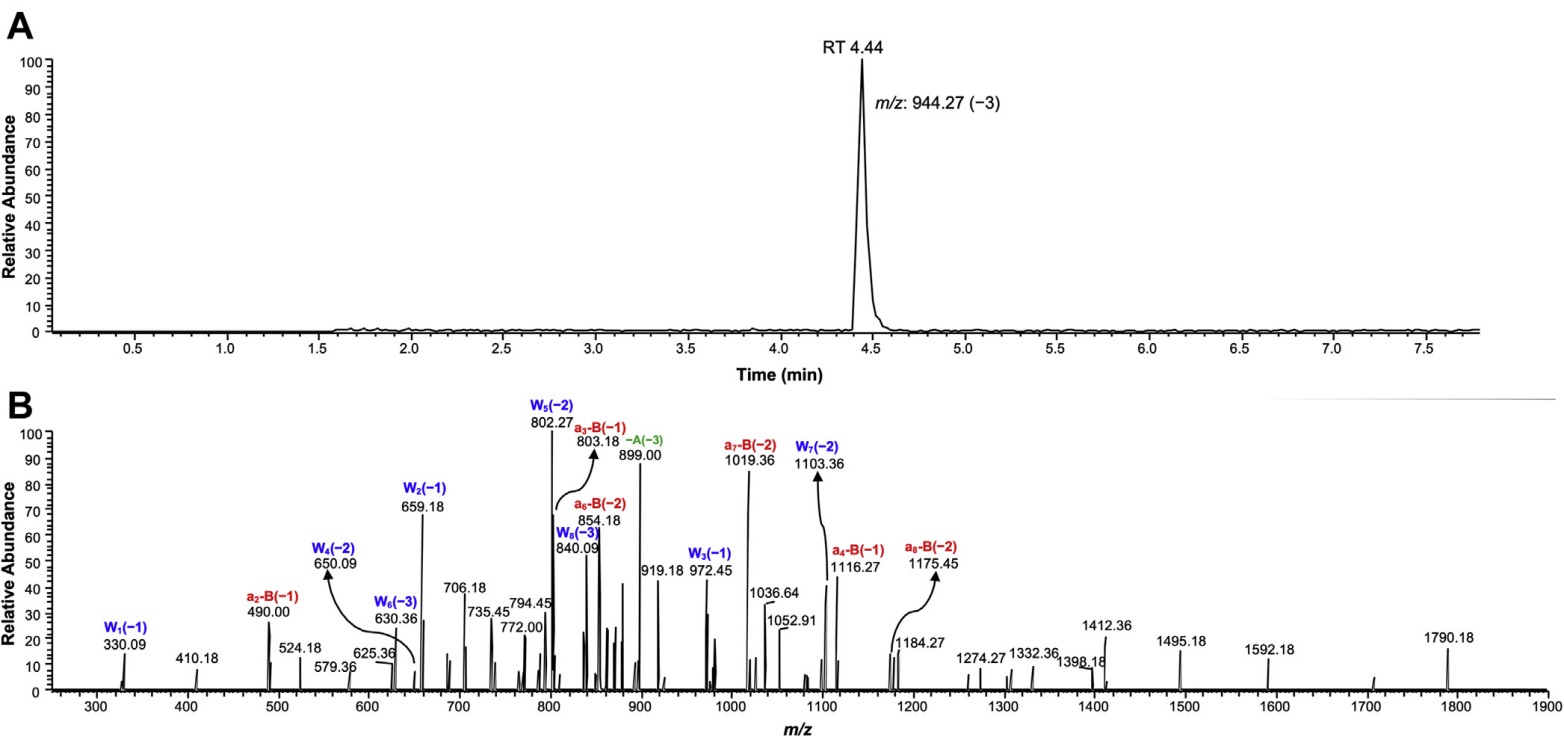
**Extracted ion chromatogram and CID spectrum of *m/z* 944.27 (−3, RT 4.44) for 15-mer peptide crosslinked to DNA**. *A*, extracted ion chromatogram; *B*, CID spectrum of *m/z* 944.27 ion.

hPol η yielded error-free products for the unmodified as well as modified template–primer complex with or without blunt end additions of A and G. For the unmodified template–primer complex, the major *m/z* 1048.64 (−3) and 1053.91 (−3) ions were observed (Figs. S13 and S14; Tables S4 and S5). For the modified template–primer complex, the major *m/z* 944.27 (−3) ion observed indicates insertion of the correct base T (Fig. 7, S15 and Table S6). In addition to this, *m/z* 1048.64 (−3) and 1053.82 (−3) were also observed, indicating insertion of the correct base T with blunt end addition of A, as well as G (Figs. S16 and S17; Tables S7 and S8). Overall, mass spectral analysis revealed only error-free bypass of the adducted peptide.

## Discussion

DNA–protein cross-links have been shown to be formed from both exogenous and endogenous sources. Protein–chemical–DNA cross-links are generally considered to be too large for DNA polymerase action (37), and it has been proposed that proteases cleave the protein to peptides. Walter's group reported the “repair” of an artificial DNA–protein cross-link by replication-coupled proteolysis in *Xenopus* egg extracts (57). DNA-dependent proteases have been identified in yeast (Wss1, Ddi1) (58), and mammalian SPRTN has been characterized (4, 16, 17, 18, 29, 59), with all three of these proteases acting on DNA–protein cross-links. These systems are operative in cultured human cells (8, 9), yeast (58), amphibians (18, 57), and plants (19). In addition to FAM111A, another mammalian protein that interacts with SPRTN (GCNA) has recently been identified, although protease activity has not been demonstrated (60, 61). Degradation by the proteasome has been presented as an alternative to direct protease action (18, 62). However, this process may require removal (from DNA) by nucleases (19, 36) and replication proceeding following the latter steps of nucleotide repair, although nuclear proteasomes and ubiquitination are known and direct proteolysis of the protein in a DNA–protein cross-link cannot be ruled out (63, 64). Human diseases have been associated with defective DNA–protein cross-link proteases (6), *e.g.*, gracile bone dysplasia (GCLEB) and Kenny–Caffey syndrome type 2 are linked to mutations in FAM111A (65, 66), pediatric germ cell tumors have been associated with mutations in GCNA (60), and Ruijs–Aalfs syndrome has been linked to mutations in SPRTN (67, 68).

DNA–peptide cross-links can be bypassed by TLS polymerases. TLS polymerases have been shown to bypass a GSH-based adduct (52, 69, 70) and (N-terminal-linked) peptides as long as 5- and 12-mers (71, 72, 73) but not a 23-mer peptide or green fluorescent protein (72). *In vitro* studies, including some in cells, have shown the mutagenic bypass of DNA–peptide cross-link by TLS polymerases (11, 42, 74). It is important to know how a DNA polymerase can bypass each cross-linked peptide entity and potentially cause base-pair mutations. As emphasized earlier, the cross-linking of a protein to DNA is not necessarily related to mutation (22, 23), and exactly why some cross-links are associated with miscoding but others are not is not understood. The reason could be lack of degradation or the inherent miscoding potential of the peptides resulting from proteolysis.

EDB induces AGT-DNA cross-links in cellular environments (Fig. 1) (48, 49, 50, 51, 75). The possibility exists that proteolytic degradation of AGT-DNA cross-links occurs under cellular conditions by DNA-dependent proteases as well as the proteasome. Therefore, we were interested in understanding the biological consequences of peptide–DNA cross-link generated from the active site of AGT, the known major site of reaction (51). Following preliminary studies indicating the bypass of GSH (3-mer) and shorter AGT-derived peptides (3-, 7-, 11-mer), we checked and focused on a 15-mer peptide cross-linked to DNA for further studies. The chemical synthesis of the DNA–peptide cross-link was achieved by coupling (Michael addition) between the dha peptide and a thiol-containing oligonucleotide (Fig. 2). An oxidized DNA–peptide cross-linked product was observed. We were unsuccessful in blocking its formation, so enzymatic and mass spectral studies were performed with this oxidized form of the DNA–peptide cross-link (Fig. 2). The same scenario was observed previously in the analysis of the DNA-AGT cross-link. Proteomic analysis of AGT-*N*^6^-DNA cross-link indicated the presence of a singly oxidized sulfur during in-gel (6.5% of oxidized product) as well as in-solution (0.8% of oxidized product) digestion by trypsin (data not shown) (43). We had not observed the presence of sulfoxides or sulfones in our analysis of AGT tryptic peptides cross-linked to DNA in previous work (50, 51), but these assays were done more rapidly than the synthetic work described here and we had set up our searches based only on the thioether product.

The full-length extension assays showed that hPol η tolerated the 15-mer peptide cross-linked to DNA, as compared with hPol ⍳ and κ (Fig. 4). Single nucleotide insertion assays indicated that only hPol η had high levels of misincorporation of each dNTP opposite to unmodified template. hPol η mediated low levels of misincorporation of all three dNTPs across from the adducted site but hPol ⍳ and κ showed only high-fidelity bypass of the adduct (Fig. 5). These results are evidence that the outcomes are not related to the oxidation of the peptide–DNA cross-link, which we have localized to the sulfur atom and not some unusual modification of the purine or deoxyribose ring.

When we compared specificity constants for incorporation of each dNTP opposite to an unmodified template, hPol η inserted dATP 28-fold, dCTP 36-fold, and dGTP 67-fold less efficiently than dTTP (Figs. 6 and S11; Table 1). Across from the peptide-adducted site, a similar trend was seen but with lower specificity constants (Table 1): hPol η inserted dATP 10-fold, dCTP 4.3-fold, and dGTP 19-fold less efficiently than dTTP. Overall, the misincorporation frequency was 37%.

LC-MS/MS analysis of hPol η bypass of the 15-mer peptide cross-linked to DNA indicated that the major product resulting from insertion had only the correct base T opposite to the lesion site (with or without blunt end addition) (Table 2). These findings are consistent with previous results showing efficient bypass of adducts in the major groove of DNA by hPol η (76, 77, 78). We conclude that other bases (A, C, G) can be inserted opposite to the adduct (Table 1) but that hPol η is not able to extend the primer to obtain full-length products. Whether or not these truncated products could be lethal to a cell is unknown at this point.

In conclusion, the steady-state kinetics showed misinsertion by hPol η while mass spectral analysis revealed only error-free product while bypassing the 15-mer peptide cross-linked to DNA. Previous studies with a number of *N*^6^-dA adducts (*e.g.*, *N*^6^-oxopropenyl-dA, *N*^6^-carboxymethyl-dA, *N*^6^-(2-hydroxy-3-butan-1,4-diyl)-dA) have not reported any mutagenic events (79, 80, 81). In the case of mutations arising from EDB, most are G to A transitions, and the numbers of A mutations we have observed in *Escherichia coli* (51, 82) yeast (83) or mouse liver (75) are too small, relative to background, to accurately assign a prominent base-pair substitution. However, hPol κ inserted C opposite to an *N*^6^-A dihydroxybutyl GSH adduct formed from diepoxybutane (70). The EDB adduct formed with the tripeptide GSH showed misincorporation of dCTP (6%) and a −1 frameshift (3%) in the LC-MS/MS analysis, as well as incorporation of mainly dGTP but also some dATP and dCTP (total ∼30%) in steady-state kinetic analysis (52). Thus, it appears that hPol η and (possibly other DNA polymerases) are capable of inserting bases opposite sites of bound peptides but the size of the peptide may be an issue in extension of mispairs (but not correct matches). Further investigation of this general hypothesis is in order.

## Experimental procedures

### Reagents

The catalytic cores of hPol η (amino acids 1–432), hPol κ (amino acids 19–526), and hPol ɩ (1–420) were expressed in *E. coli* and purified as reported previously (84, 85, 86). Unlabeled dNTPs and UDG were purchased from New England Biolabs (Ipswich, MA). C_18_ Sep-Pak columns were purchased from Waters (Milford, MA). Micro Biospin-6 columns were purchased from Bio-Rad. Unmodified oligonucleotide and FAM-labeled DNA primers (HPLC-purified) were purchased from Integrated DNA technologies (Coralville, IA). The 6-chloropurine phosphoramidite was synthesized by Professor Carmelo Rizzo (Vanderbilt University, Nashville, TN), and an oligonucleotide containing the 6-chloropurine residue was synthesized as previously reported (52). The 15-mer peptide was purchased from New England Peptides (Gardner, MA).

### Synthesis of MSH (87)

Ethyl hydroxamate (23 mmol) was stirred in a 2:1 (v/v) mixture of DMF and triethylamine (9 ml) and cooled to 0 °C. Mesitylene sulfonyl chloride (23 mmol) was slowly added, and the reaction was stirred for an additional 15 min before adding CH_2_Cl_2_ (100 ml). The reaction product was washed ten times with H_2_O, and the organic layer was collected and dried using brine and MgSO_4_ before removing the solvent in vacuo. The reaction product was redissolved in dioxane (4 ml) and cooled to 0 °C before adding 70% HClO_4_ dropwise over 2 min (1.8 ml). The solution was stirred for an additional 2 min and transferred into chilled H_2_O (200 ml) prior to extraction with diethyl ether (100 ml). The organic layer was neutralized, dried using K_2_CO_3_, and filtered. The filtrate was concentrated, and chilled hexane (150 ml) was added to crystalize the product overnight at −20 °C. MSH crystals were collected, dried in a vacuum desiccator, and stored at −20 °C.

### Synthesis of 15-mer dehydroalanine peptide (dha peptide)

K_2_CO_3_ (9 mg) was dissolved in 100 μl of H_2_O and added to the 15-mer peptide (acyl-PVPILIPCHRVVSSS-amide). MSH (3.8 mg) was dissolved in 100 μl of anhydrous DMF and added dropwise into the peptide solution followed by incubation on ice for 20 min. After completion of the reaction, the reaction mixture was diluted in 800 μl of Mobile Phase A (95% H_2_O, 5% CH_3_CN, and 0.1% HCO_2_H, v/v/v) and purified by HPLC using a Phenomenex semipreparative octadecylsilane (C_18_) HPLC column (10 mm × 250 mm, 5 μm). The sample was eluted at a flow rate of 3 ml min^−1^ (UV detection at 240 nm). Buffers consisted of Mobile Phases A and B (95% CH_3_CN, 5% H_2_O, 0.1% HCO_2_H, v/v/v). The following gradient was used: 0–5 min, 10% B; 5–20 min, 10–50% B; 20–25 min, 50–100% B; 25–30 min, 100% B and 30–32 min, 100–10% B (all v/v). The 15-mer dha peptide eluted at approximately 19.7 min (Fig. S1), and the fraction was collected and lyophilized. The mass was confirmed (Figs. S2 and S3, Table S2) by ESI-MS analysis (using a Finnigan LTQ mass spectrometer (Thermo Scientific, San Jose, CA)), acquired in the ESI positive mode following introduction from an octdecylsilane C_18_ column (Acquity UPLC BEH, 1.7 μm, 2.1 mm × 50 mm). Buffers consisted of Mobile Phases A (95% H_2_O, 5% CH_3_CN, and 0.1% HCO_2_H) and B (95% CH_3_CN, 5% H_2_O, and 0.1% HCO_2_H), all v/v/v. The temperature of the column was 40 °C. Product ion spectra were collected over the *m/z* range of 150–2000, followed by MS/MS fragmentation of the *m/z* 806.5 and 823.5 ions. The MS conditions were as follows: voltage 5 kV, sheath gas 50, auxiliary gas 35, sweep gas 5, capillary voltage 13 V, capillary temperature 350 °C, tube lens 60 V, full MS and MS^n^ target ion count 1e4, full MS, and MS^n^ max injection time 200 ms.

### Synthesis of N^6^-cystamine-dA oligonucleotide

The controlled pore glass (CPG, 1 μmol) of the 19-mer oligonucleotide (5′-TCTCXGTTTATGGACCACC-3′, where X is 6-Cl purine) was treated with cystamine·2HCl [10 μl of 500 mM stock in dimethylsulfoxide (DMSO)] in a mixture of DMSO (86 μl) and diisopropylethylamine (4.4 μl) to obtain the *N*^6^-cystamine-dA modified oligonucleotide. After incubation at 55 °C for 22 h, the supernatant was removed. The oligonucleotide (still attached to the glass support) was washed with anhydrous DMSO (2 × 1 ml) and anhydrous CH_3_CN (3 × 1 ml) and then air-dried. The oligonucleotide was subjected to final deprotection using 0.4 M NaOH in CH_3_OH (1 ml) at room temperature for 17 h, with shaking. The oligonucleotide support was sonicated for 5 min, and the supernatant was collected in an Eppendorf tube. The oligonucleotide support was washed with H_2_O (2 × 400 μl), and the supernatant was collected in the same tube. The collected supernatant was neutralized to pH 7.0 using glacial CH_3_CO_2_H and concentrated *in vacuo* using a centrifugal evaporator. The dried pellet was resuspended in H_2_O (500 μl) for HPLC purification. Purification was carried out using a Phenomenex Clarity Oligo RP (C_18_) column (150 mm × 110 mm, 5 μm). The oligonucleotide was eluted at a flow rate of 3 ml min^−1^ (UV detection at 260 nm). Buffers consisted of Mobile Phases A (0.1 M triethylammonium acetate, pH 7.0) and B (0.1 M triethylammonium acetate in 50% CH_3_CN, v/v). The following gradient was used: 0–5 min, 17% B; 5–20 min, 17–40% B; 20–21 min, 40%–100% B; 21–26 min, 100% B; 26–27 min, 100–17% B; and 27–30 min, 17% B (all v/v). The desire oligonucleotide eluted at 13.6 min (Fig. S4). The oligonucleotide fraction was collected and concentrated *in vacuo* with a centrifugal evaporator and resuspended in 10 mM Tris-HCl buffer (pH 8.0), 1 mM EDTA, and 300 mM NaCl. The modified oligonucleotide was desalted using C_18_ Sep-Pak, and the mass was confirmed by MALDI-TOF (Fig. S5).

### Synthesis of 15-mer peptide crosslinked at N^6^-dA position

To the *N*^6^-cystamine-dA oligonucleotide (3 nmol, in 44 μl nuclease free water), 10 mM potassium phosphate buffer (pH 8.0) and 2 mM DTT were added to obtain an *N*^6^-(2-thioethyl)-dA modified oligonucleotide (Scheme S2). The reaction was incubated at 95 °C for 5 min and cooled to room temperature. The reaction mixture was desalted by passing through a Micro Bio-Spin chromatography column following the manufacturer's protocol. The dha peptide (1 mg) was dissolved in 10% CH_3_CN (v/v, 40 μl). The peptide solution was added to *N*^6^-(2-thioethyl)-dA modified oligonucleotide (Fig. 2), and reaction mixture was incubated at 37 °C for overnight. Details of the alternative synthetic route can be found in the reported procedure (55). The cross-linking reaction mixture was purified using gel electrophoresis (Fig. S6). The desired cross-link band was visualized using a UV lamp, cut out of the gel, and extracted using 10 mM Tris-HCl buffer (pH 8.0) containing 1 mM EDTA and 300 mM NaCl. The DNA–peptide cross-link was desalted using a C_18_ Sep-Pak, and the mass was confirmed by MALDI as well as negative ion ESI-MS (Figs. 3 and S7). An additional mass of 32 a. m. u. was found.

The sample was further analyzed following hydrolysis of the DNA entity with HF to obtain a peptide adducted with adenine (Fig. S8). The cross-link (100 pmol) was dried and suspended in HF (48%, 50 μl), and incubated at 4 °C for 14 h (43). The sample was dried under a stream of nitrogen, resuspended in anhydrous CH_3_OH (50 μl), and dried again under a stream of nitrogen. Finally, the digested cross-link was dissolved in 0.1% HCO_2_H (20 μl, v/v) and shaken for 10 min. Following centrifugation for 5 min at 21,000*g* at room temperature, the crude sample was directly (without any purification) analyzed in the positive mode using nano LC-ESI-MS (Figs. S9 and S10, Table S3). The fragmentation is consistent with the additional mass of 32 a. m. u. being on the linker between the peptide and the oligonucleotide.

### Full-length extension assays

A FAM-labeled 12-mer DNA primer (5′-FAM-GGTGGTCCATAA-3′) and a 19-mer DNA template (5′-TCTCXGTTTATGGACCACC-3′, where X is dA or the *N*^6^-dA-peptide) were annealed (1:1 M ratio) at 95 °C for 5 min and cooled to room temperature overnight. Reactions were carried out using 50 mM Tris-HCl buffer (pH 7.5) containing 5% glycerol (v/v), 5 mM DTT, 50 mM NaCl, 5 mM MgCl_2_, 50 μg ml^−1^ BSA, 120 nM primer–template complex, and 250 μM dNTPs. In order to obtain fully extended products, either 20 nM of hPol η, or 40 nM hPol ⍳, or 20 nM hPol κ was used. Reactions were initiated by adding a 1 μl mixture of dNTPs to a total volume of 25 μl. Aliquots of 3 μl of the reaction mixture were removed at different time points (0, 5, 10, 20, 60, and 120 min) and quenched with 7.5 μl of 10 mM EDTA (pH 8.0) in 95% deionized formamide. Products were separated using PAGE (Fig. 4), and results were analyzed by ImageJ software by visualizing on a Typhoon scanner (GE Healthcare).

### Single nucleotide insertion assays

A FAM-labeled 14-mer primer (5′-FAM-GGTGGTCCATAAAC-3′) and a 19-mer template (5′-TCTCXGTTTATGGACCACC-3′, where X is dA or *N*^6^-dA-peptide) were annealed (1:1 M ratio) at 95 °C for 5 min and cooled to room temperature overnight. Reactions were carried out using 40 mM Tris-HCl buffer (pH 7.5) containing 5% glycerol (v/v), 10 mM DTT, 100 mM KCl, 5 mM MgCl_2_, 0.1 mg ml^−1^ BSA, 120 nM primer–template complex, and 100 μM each individual dNTP. Independent reactions were done for each nucleotide. Enzyme concentrations of 5 nM of hPol η, 5 nM hPol ⍳, and 5 nM hPol κ were used to obtain mainly single nucleotide insertions. Reactions were started by adding 1 μl of each dNTP to a total volume of 10 μl. Aliquots of 2 μl of the reaction mixture were removed at different time points (0, 5, 10, and 30 min) and quenched with 7.5 μl of 10 mM EDTA (pH 8.0) in 95% deionized formamide. Products were separated using PAGE (Fig. 5), and results were analyzed by ImageJ software by visualization with a Typhoon scanner.

### Steady-state kinetics

A FAM-labeled 14-mer primer (5′-FAM-GGTGGTCCATAAAC-3′) and a 19-mer template (5′-TCTCXGTTTATGGACCACC-3′, where X is dA or *N*^6^-dA-peptide) were annealed (1:1 M ratio) at 95 °C for 5 min and cooled to room temperature overnight. Reactions were carried out using 40 mM Tris-HCl buffer (pH 7.5) containing 5% glycerol (v/v), 10 mM DTT, 100 mM KCl, 5 mM MgCl_2_, and 0.1 mg ml^−1^ BSA. Varying concentrations of each dNTP were used to achieve ≤20% of product formation, with up to 11 different concentrations of each dNTP were used. Reactions were started by adding 1 μl of individual dNTP stock solutions. Aliquots of 3 μl were removed at different time points (depending on the polymerase, template, and dNTP) and quenched with 6.5 μl of 10 mM EDTA (pH 8.0) in 95% deionized formamide. Products were separated using PAGE (Figs. 6 and S11), and results were analyzed by ImageJ software by visualizing on a Typhoon scanner. Reactions were done in duplicates. Data points are shown as means (±SD) and estimated using fit to a hyperbolic equation in Prism (GraphPad, San Diego, CA) software to calculate the *k*_cat_ and *k*_cat_/*K*_m_ (*k*_sp_), then deriving *K*_m_ (Table 1) (88).

### LC-MS/MS analysis

A 12-mer primer containing a 2′-deoxyuridine (5′-FAM-GGTGGTCCA**U**AA-3′) was annealed to the 19-mer, with modified as well as unmodified templates (5′-TCTCXGTTTATGGACCACC-3′), in a 1:1 M ratio at 95 °C for 5 min and cooled to room temperature overnight. Full-length extension reactions were carried out using 50 mM Tris-HCl buffer (pH 7.5) containing 5% glycerol (v/v), 5 mM DTT, 50 mM NaCl, 5 mM MgCl_2,_ and 50 μg ml^−1^ BSA, hpol η (0.75 μM), primer–template complex (2.5 μM), and a mixture of all four dNTPs (1 mM). Full-length extension reactions were carried out at 37 °C for 4 h. After completion, reactions were quenched by removing Mg^2+^ and dNTPs using spin columns. Extension products were treated with UDG (25 U) for 4 h at 37 °C followed by treatment with piperidine (0.25 M) for 1 h at 95 °C (Fig. S12). After 1 h, nuclease-free water (300 μl) was added to it and the reaction mixtures were frozen, lyophilized, and then azeotroped by repeating the same step. The product was dissolved in nuclease-free water (30 μl) and analyzed (Figs. 7 and S13–S17; Tables 2 and S4–S8) in the negative ion mode on Waters Acquity UPLC system attached to a Thermo-Finnigan LTQ mass spectrometer (electrospray ionization), as described previously (89).

## Data availability

All data are included in the article and supporting information.

## Supporting information

This article contains supporting information.

## Conflict of interest

The authors declare that they have no conflict of interest with the contents of this article.

## Supporting information

The supporting information contains a list of oligonucleotide and peptide sequences used in this study; synthetic strategies of dehydroalanine peptide and *N*^6^-cystamine-dA modified oligonucleotide; HPLC purification, mass spectrum, and fragmentation pattern of dehydroalanine peptide; MALDI spectrum of *N*^6^-cystamine-dA modified oligonucleotide; gel purification, mass spectrum of peptide–DNA cross-link; flow chart of HF treatment, mass spectrum, and fragmentation pattern of peptide–DNA cross-link; PAGE analyses of steady-state kinetics and the Prism graphs; LC-ESI-MS/MS sequencing analysis of full-length extension reaction: extracted ion chromatograms and CID fragmentation spectrums.

Schemes S1 and S2; Figures S1 to S17; Tables S1 to S8
