# Supplementary material for: Enzymatic bypass of an N6-deoxyadenosine DNA–ethylene dibromide–peptide cross-link by translesion DNA polymerases
Source: J Biol Chem. 2021 Feb 20;296:100444. doi: 10.1016/j.jbc.2021.100444 (PMC8024977; doi:10.1016/j.jbc.2021.100444)
Supplement: Schemes S1 and S2; Figures S1 to S17; Tables S1 to S8 [file mmc1.pdf]

SUPPORTING INFORMATION

Enzymatic bypass of an *N*[6]-deoxyadenosine DNA-ethylene dibromide-peptide crosslink by translesion DNA polymerases

Pratibha P. Ghodke,[1] Gabriela Gonzalez Vasquez,[1] Hui Wang, Kevin M. Johnson, Carl A. Sedgeman, and F. Peter Guengerich*

Department of Biochemistry, Vanderbilt University School of Medicine, Nashville, Tennessee 37232-0146

E-mail: f.guengerich@vanderbilt.edu

[1]These authors contributed equally to this work and should be considered co-first authors.

Contents

**Table S1**	List of oligonucleotide and peptide sequences	Page S3
**Scheme S1**	Synthetic strategy for dehydroalanine-modified 15-mer peptide	Page S3
**Figure S1**	HPLC purification of 15-mer dehydroalanine peptide	Page S4
**Figure S2**	LC-ESI-MS/MS analysis of dehydroalanine 15-mer peptide	Page S4
**Figure S3**	Extracted ion chromatogram and CID spectrum of *m/z* 806.09 (+2) of 15-mer dehydroalanine peptide	Page S5
**Table S2**	Observed and theoretical CID fragments of *m/z* 806.09 for dehydroalanine modified peptide	Page S6
**Scheme S2**	Synthetic strategy for N^6-cystamine-dA-modified oligonucleotide	Page S6
**Figure S4**	HPLC purification of N^6-cystamine-dA-modified oligonucleotide	Page S7
**Figure S5**	MALDI spectrum of N^6-cystamine-dA-modified oligonucleotide	Page S8
**Figure S6**	Gel purification of N^6-dA DNA-peptide crosslink	Page S8
**Figure S7**	ESI-MS characterization of N^6-dA-peptide crosslink	Page S9
**Figure S8**	Flow chart for HF treatment	Page S9
**Figure S9**	LC-ESI-MS/MS analysis of HF treated N^6-dA-peptide crosslink	Page S10
**Figure S10**	Extracted ion chromatogram and CID spectrum of *m/z* 613.66 (−3) of HF treated N^6-dA-peptide crosslink	Page S10
**Table S3**	Observed and theoretical CID fragments of *m/z* 613.66 of HF treated N^6-dA-peptide crosslink	Page S11
**Figure S11**	Steady-state kinetic analysis of dATP, dCTP, and dGTP insertion by hPol η	Page S12
**Figure S12**	PAGE of full-length extension reactions, UDG and piperidine treatment	Page S13
**Figure S13**	Extracted ion chromatogram and CID spectrum of *m/z* 1048.64 (−3) for control template	Page S13
**Table S4**	Observed and theoretical CID fragments of *m/z* 1048.64 (−3) for control template	Page S14
**Figure S14**	Extracted ion chromatogram and CID spectrum of *m/z* 1053.91 (−3) for control template	Page S15
**Table S5**	Observed and theoretical CID fragments of *m/z* 1053.91 (−3) for control template	Page S16
**Figure S15**	Extracted ion chromatogram *m/z* 944.27 (−3) and observed peak *m/z* 1048.68 (−3) for peptide-DNA crosslink	Page S17
**Table S6**	Observed and theoretical CID fragments of *m/z* 944.27 (−3) for peptide-DNA crosslink	Page S17
**Figure S16**	Extracted ion chromatogram and CID spectrum of *m/z* 1048.64 (−3) for peptide-DNA crosslink	Page S18
**Table S7**	Observed and theoretical CID fragments of *m/z* 1048.64 (−3) for peptide-DNA crosslink	Page S18
**Figure S17**	Extracted ion chromatogram and CID spectrum of *m/z* 1053.82 (−3) for peptide-DNA crosslink	Page S19
**Table S8**	Observed and theoretical CID fragments of *m/z* 1053.82 (−3) peptide-DNA crosslink	Page S19

Table S1

List of oligonucleotide and peptide sequences

Code	Oligonucleotide and peptide sequences	Calculated [M + H]$^+$	Observed [M + H]$^+$
15-mer Peptide	Acyl-PVPILIPCHRVVSSS-amide		
15-mer Dehydroalanine (dha) peptide	Acyl-PVPILIPdhaHRVVSSS-amide	1610.93	1611.00
N^6-Cystamine-dA oligonucleotide	5′-TCTCXGTTTATGGACCACC-3′	5878.0317	5876.2090
15-mer Peptide crosslinked to *N*^{6}-dA in DNA	5′-TCTCXGTTTATGGACCACC-3′ Oxidized form	7443.8107	7443.6817
19-mer Template	5′-TCTCAGTTTATGGACCACC-3′		
12-mer Primer	5′-FAM-GGTGGTCCATAA-3′		
14-mer Primer	5′-FAM-GGTGGTCCATAAAC-3′		
12-mer Primer for LC-ESI-MS/MS	5′-FAM-GGTGGTCCA**U**AA-3′		

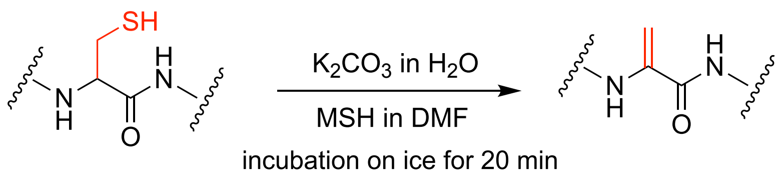

Scheme S1. Synthetic strategy for dehydroalanine 15-mer peptide

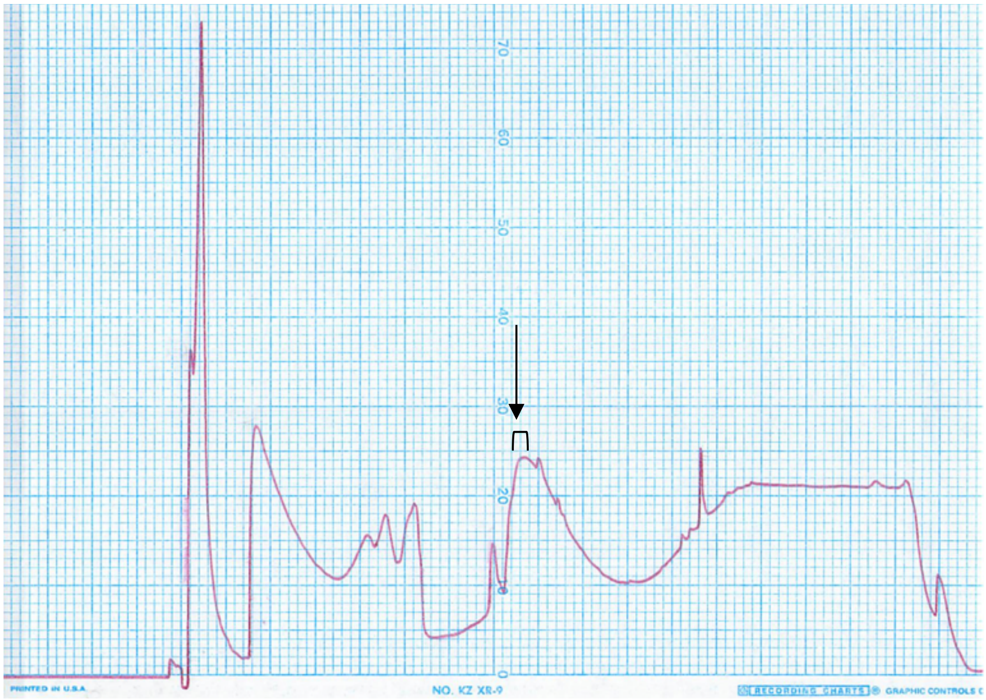

Figure S1. HPLC purification of dehydroalanine 15-mer peptide. The molecule of interest eluted at approximately 19.7 min.

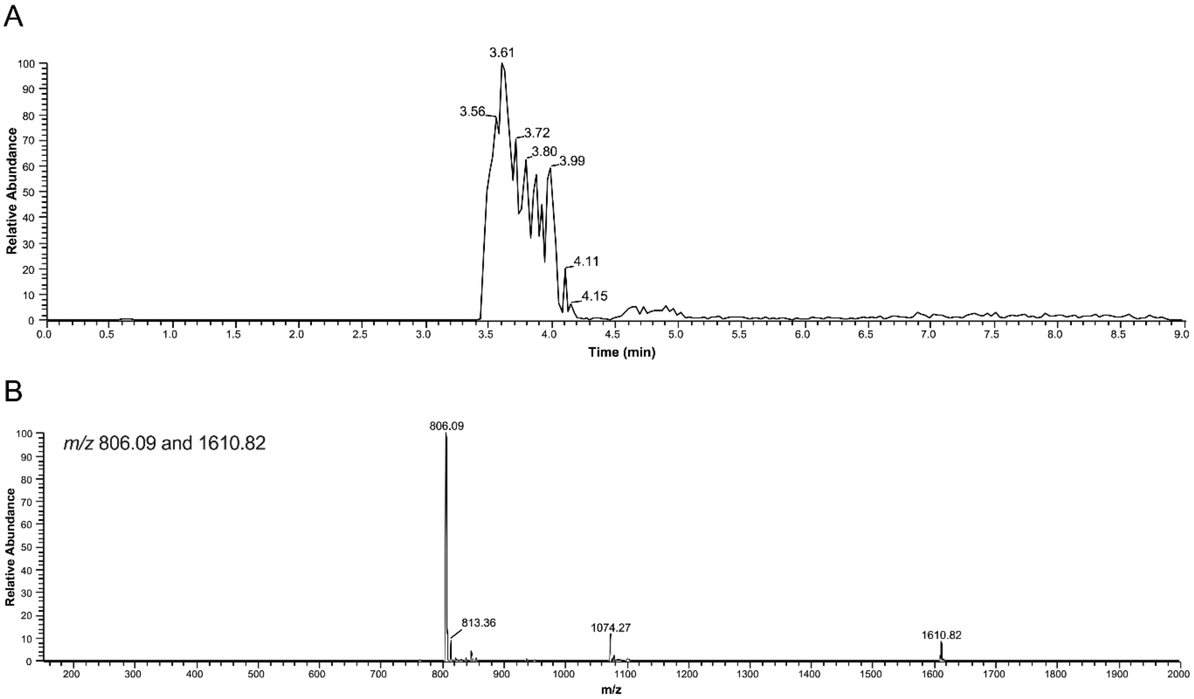

Figure S2. LC-ESI-MS analysis of dehydroalanine 15-mer peptide. *A*, total ion chromatogram. t_R 3.61 min. *B*, observed peaks at *m/z* 806.09 (+2) and 1610.82 (+1).

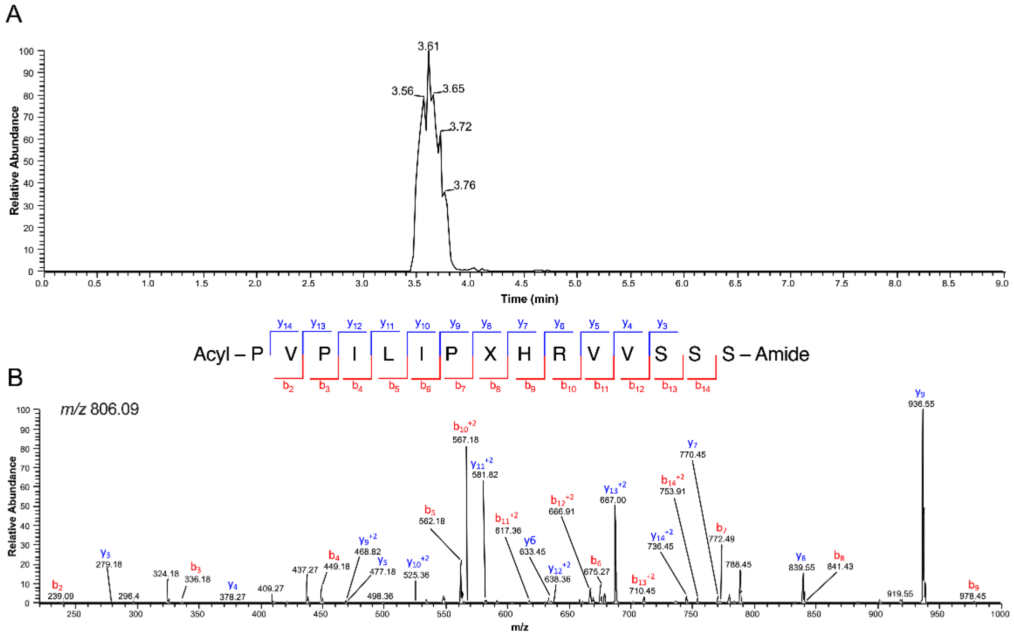

Figure S3. LC-ESI-MS/MS analysis of synthesized dehydroalanine 15-mer peptide. *A*, extracted ion chromatogram for *m/z* 806.09 (+2, t_R 3.61 min). *B*, CID spectrum for *m/z* 806.09 (+2). See Table S2 for fragment assignment. b-ions represented in red color and y-ions represented in blue color. X, dehydroalanine.

Table S2

Observed and theoretical CID fragments of *m/z* 806.09 for dehydroalanine-modified peptide. X, dehydroalanine.

Fragment assignment	*m/z* theoretical	*m/z* observed
Acyl-PV (b_2, +1)	239.14	239.09
Acyl-PVP (b_3, +1)	336.19	336.18
Acyl-PVPI (b_4, +1)	449.28	449.18
Acyl-PVPIL (b_5, +1)	562.36	562.18
Acyl-PVPILIP (b_6, +1)	675.44	675.27
Acyl-PVPILIPX (b_7, +1)	772.49	772.49
Acyl-PVPILIPXH (b_8, +1)	841.43	841.43
Acyl-PVPILIPXHR (b_9, +1)	978.49	978.45
Acyl-PVPILIPXHRV (b_{10}, +2)	567.79	567.18
Acyl-PVPILIPXHRVV (b_{11}, +2)	617.33	617.36
Acyl-PVPILIPXHRVVS (b_{12}, +2)	666.87	666.91
Acyl-PVPILIPXHRVVSS (b_{13}, +2)	710.38	710.45
Acyl-PVPILIPXHRVVSSS (b_{14}, +2)	753.89	753.91
SSS-Amide (y_3, +1)	279.13	279.18
VSSS-Amide (y_4, +1)	378.19	378.27
VVSSS-Amide (y_5, +1)	477.27	477.18
RVVSSS-Amide (y_6, +1)	633.37	633.45
HRVVSSS-Amide (y_7, +1)	770.43	770.45
XHRVVSSS-Amide (y_8, +1)	839.36	839.55
PXHRVVSSS-Amide (y_9, +1)	939.41	939.55
IPXHRVVSSS-Amide (y_{10}, +2)	525.25	525.36
LIPXHRVVSSS-Amide (y_{11}, +2)	581.79	581.82
ILIPXHRVVSSS-Amide (y_{12}, +2)	638.34	638.36
PILIPXHRVVSSS-Amide (y_{13}, +2)	686.86	687.00
VPILIPXHRVVSSS-Amide (y_{14}, +2)	736.39	736.45

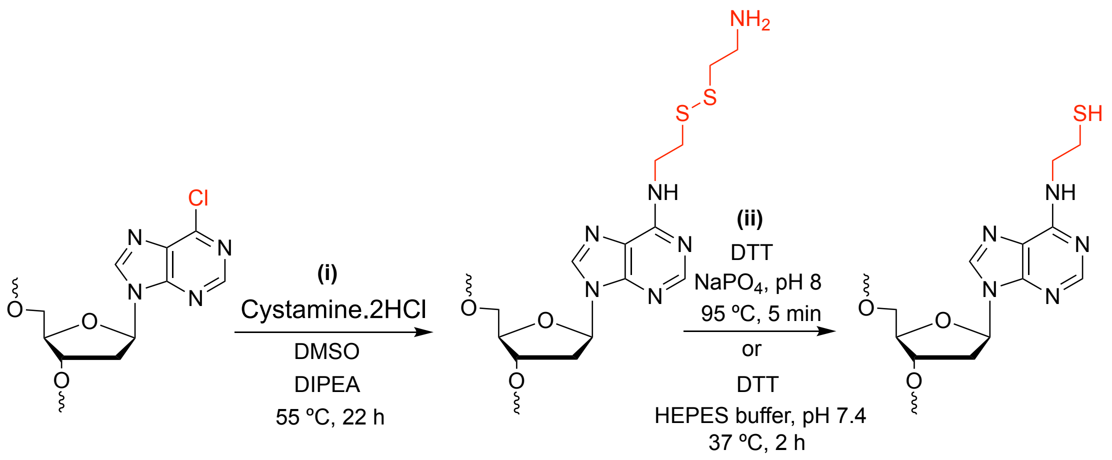

Scheme S2. Synthetic strategy for *N*⁶-cystamine-dA-modified oligonucleotide followed by reduction to obtain *N*⁶-(2-thioethyl)-dA modified oligonucleotide.

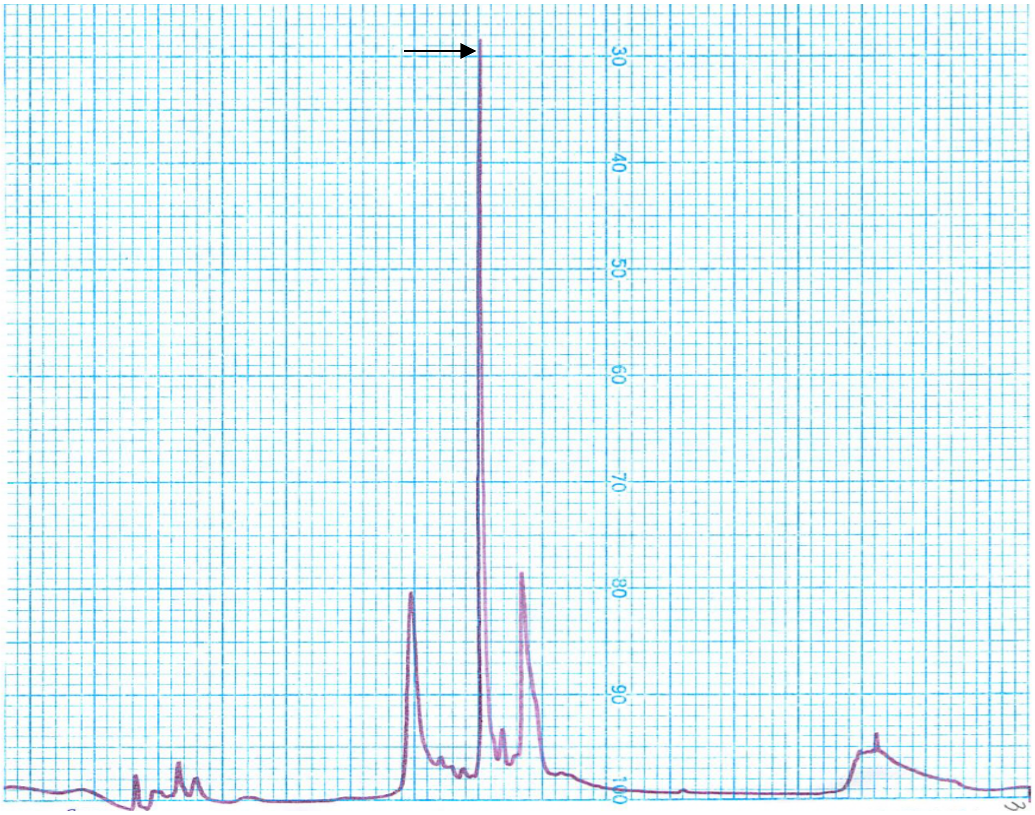

Figure S4. HPLC purification of *N*[6]-cystamine-dA-modified oligonucleotide. The oligonucleotide of interest (shown with arrow) eluted at approximately 13.6 min (marked with arrow).

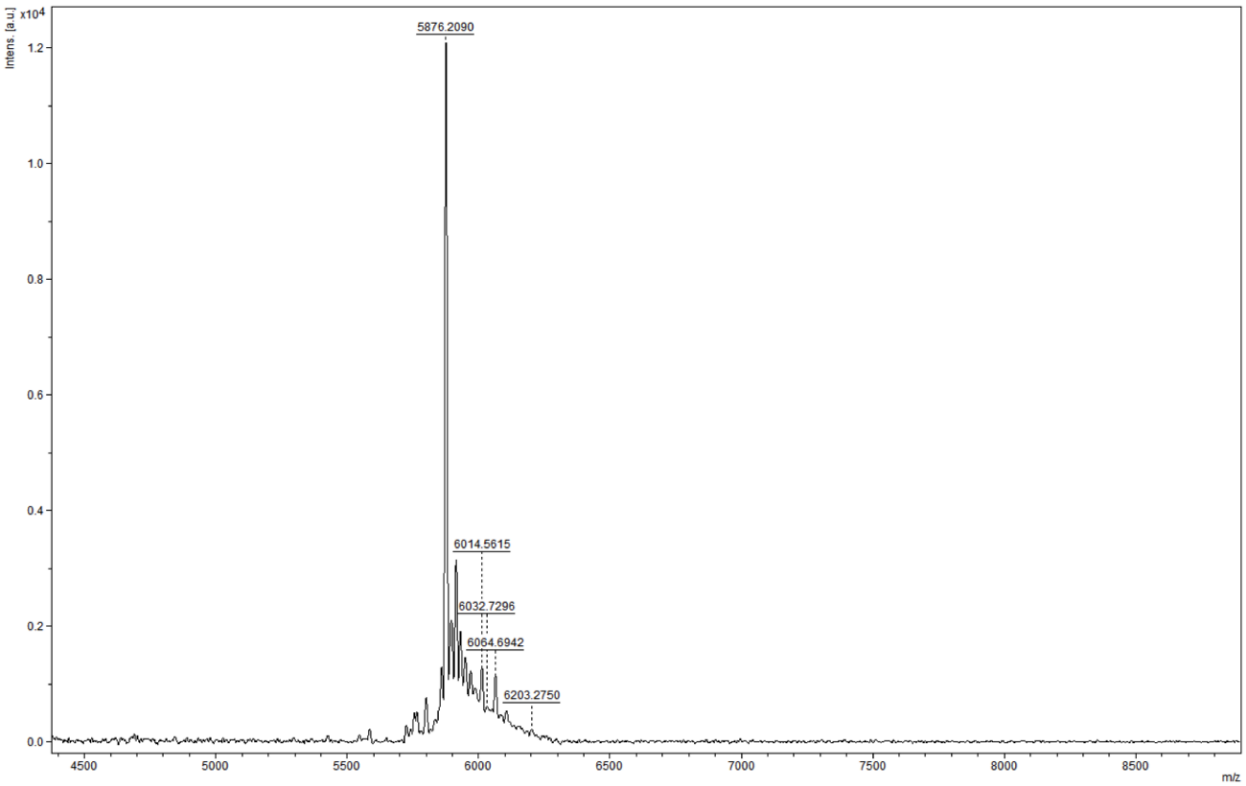

Figure S5. MALDI spectrum of N^6-cystamine-dA-modified oligonucleotide. Expected $[M + H]^+$ 5878.0317, mass $[M + H]^+$ 5876.2090.

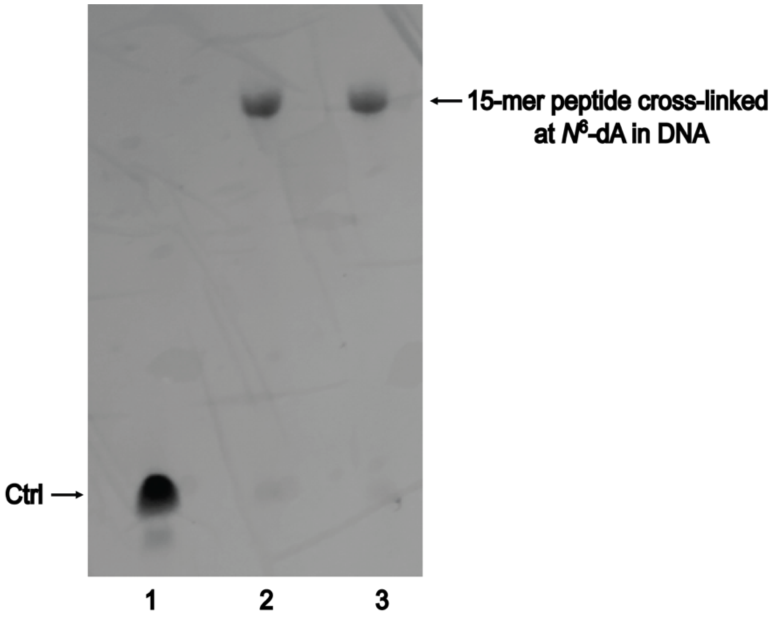

Figure S6. Gel purification (PAGE, 20%, 7 M urea) of N^6-dA-peptide crosslink. Lane 1: 19-mer DNA template. Lanes 2 and 3: Crosslinked reaction mixture.

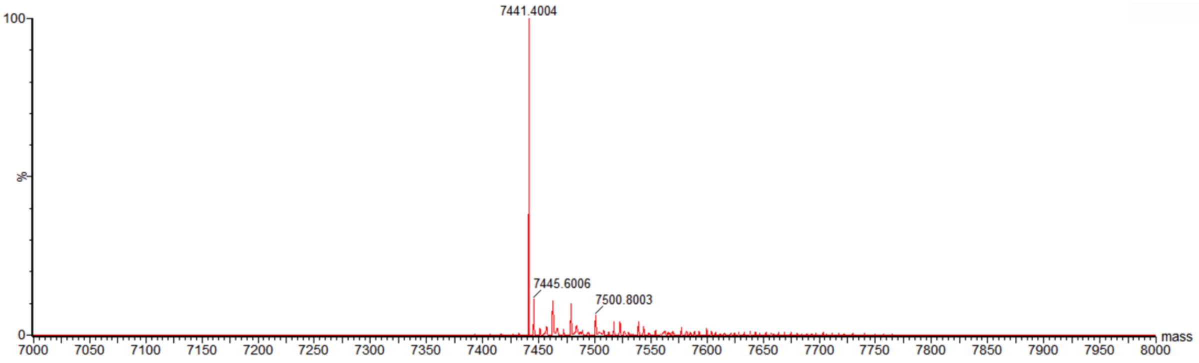

Figure S7. ESI-MS characterization of 15-mer peptide crosslinked to $\boldsymbol{N^6}$**-dA.** Expected $[M - H]^-$ 7439.8107, observed $[M - H]^-$ 7441.4004.

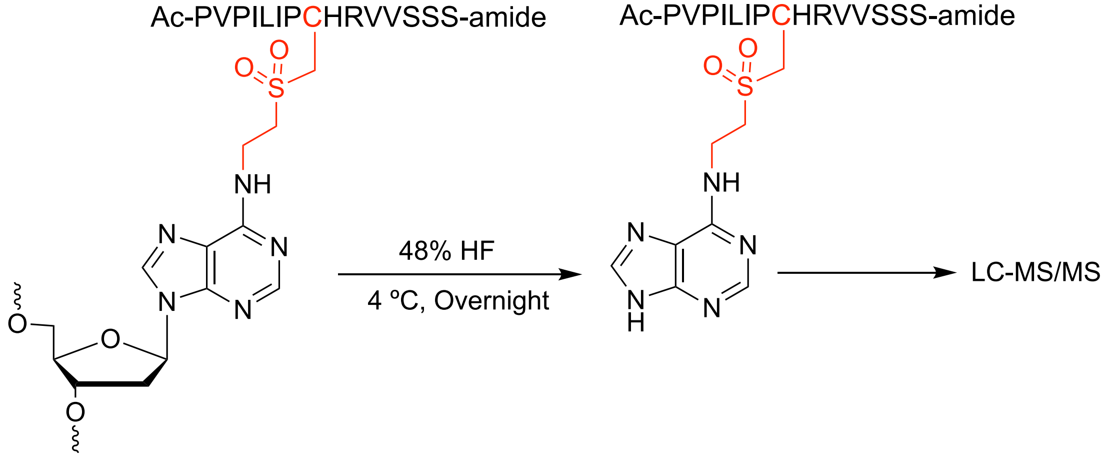

Figure S8. Hydrolysis with HF.

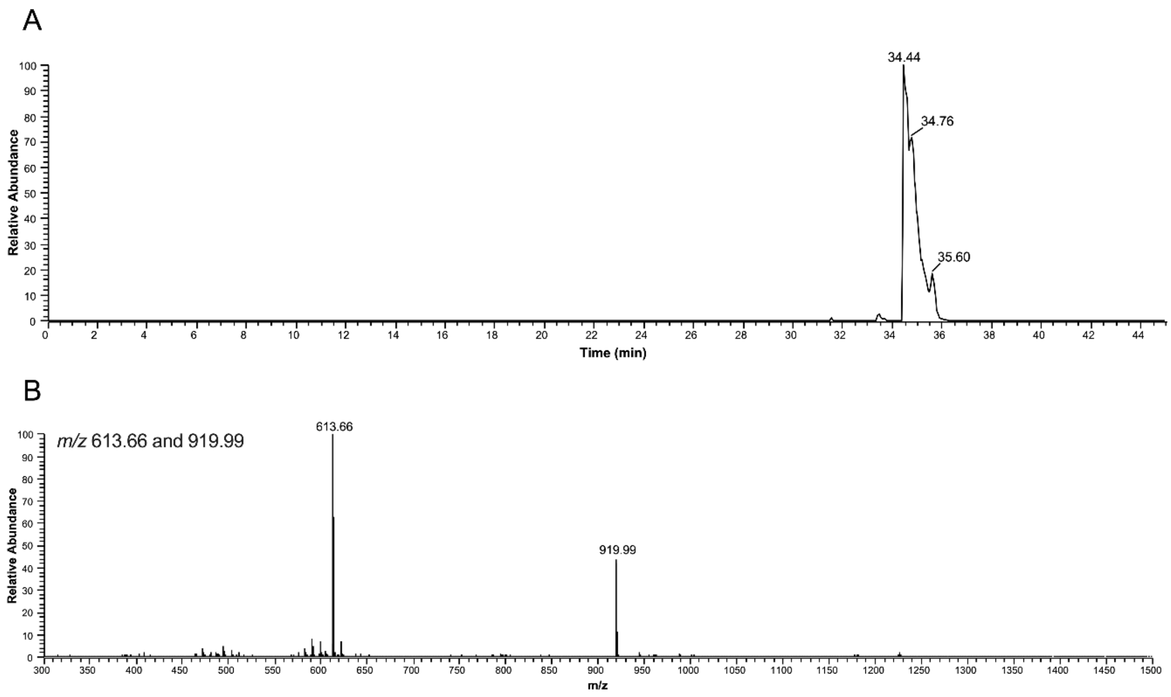

Figure S9. LC-ESI-MS/MS analysis of HF-treated 15-mer peptide crosslinked to *N*[6]-dA. *A*, extracted ion chromatogram for *m/z* 613.66 (+3, t_R 34.44 min). *B*, observed peaks at *m/z* 613.66 (+3) and 919.99 (+2).

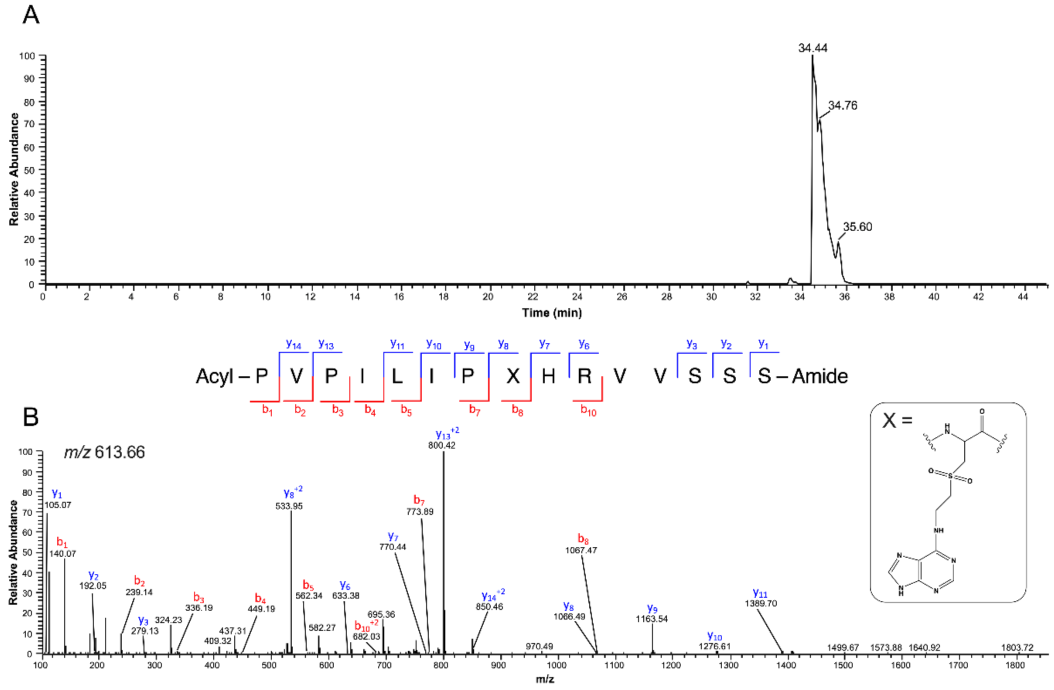

Figure S10. LC-ESI-MS/MS analysis of HF-treated 15-mer peptide crosslinked to *N*[6]-dA. *A*, Extracted ion chromatogram for *m/z* 613.66 (+3, t_R 34.4 min), same as in Fig. S9*A*. *B*, CID spectrum for *m/z* 613.66 (+3). See Table S3 for fragment assignments. b-ions represented in red color and y-ions represented in blue. X, cysteine link to A.

Table S3
Observed and theoretical CID fragments of *m/z* 613.66 for HF treated 15-mer peptide crosslinked to *N*^{6}-dA

Fragment assignment	*m/z* theoretical	*m/z* observed
Acyl-P (b1, +1)	140.07	140.07
Acyl-PV (b_2, +1)	239.14	239.14
Acyl-PVP (b_3, +1)	336.19	336.19
Acyl-PVPI (b_4, +1)	449.27	449.19
Acyl-PVPIL (b_5, +1)	562.36	562.34
Acyl-PVPILIPX (b_7, +1)	772.49	773.89
Acyl-PVPILIPXH (b_8, +1)	1068.68	1067.47
Acyl-PVPILIPXHRV (b_{10}, +2)	681.42	682.03
S-Amide (y_1, +1)	105.07	105.07
SS-Amide (y_2, +1)	192.09	192.05
SSS-Amide (y_3, +1)	279.13	279.13
RVVSSS-Amide (y_6, +1)	633.37	633.38
HRVVSSS-Amide (y_7, +1)	770.43	770.44
XHRVVSSS-Amide (y_8, +1)	1066.61	1066.49
XHRVVSSS-Amide (y_8, +2)	533.81	533.95
PXHRVVSSS-Amide (y_9, +1)	1163.66	1163.54
IPXHRVVSSS-Amide (y_{10}, +1)	1276.74	1276.61
LIPXHRVVSSS-Amide (y_{11}, +1)	1389.83	1389.70
PILIPXHRVVSSS-Amide (y_{13}, +2)	800.48	800.42
VPILIPXHRVVSSS-Amide (y_{14}, +2)	850.02	850.46

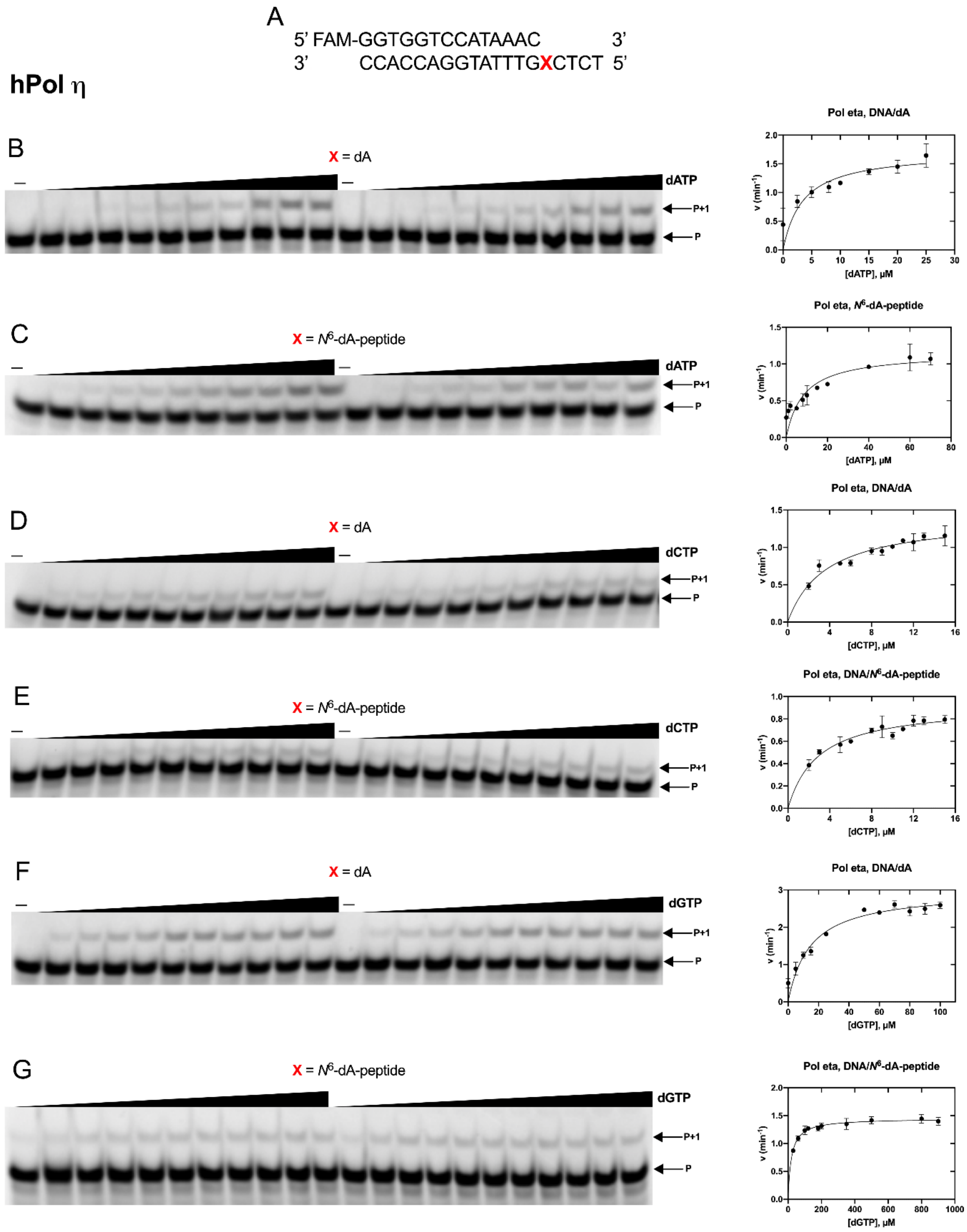

Figure S11. Steady-state kinetic analysis of dATP, dCTP, and dGTP insertion by hPol η. *A*, 14-mer primer and 19-mer template sequences, where X is dA or *N*6-dA-peptide. Reactions were done at 37 °C using hPol η (2.5 nM (*C, E*), 2 nM (*B, D*), 1.5 nM (*G*), and 0.9 nM (*F*)). Varying concentrations of dATP (0-25 μM (*B*) and 0-70 μM (*C*)), dCTP (0-15 μM (*D, E*)) and dGTP (0-100 μM (F) and 30-900 μM (G)). Reactions were carried out for 8 (F, G) and 10 (*B, C, D, E*) min.

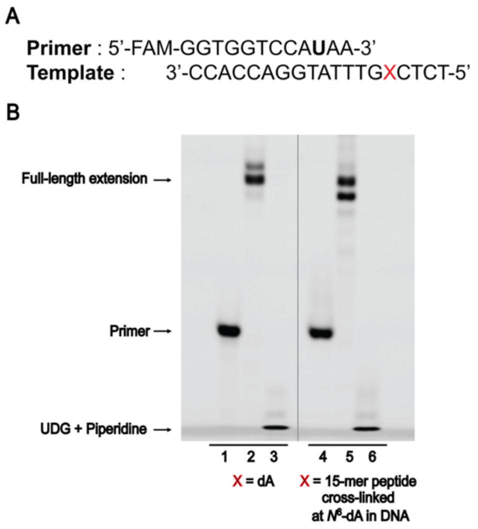

Figure S12. PAGE (20%, 7 M urea) of full-length extension reactions employing hpol η (1.4 µM) in the presence of a mixture of dNTPs. *A*, primer-template complex; *B*, full-length extension reactions for unmodified as well as cross-linked template-primer complex (Lane 1 and 4 included FAM-labeled dU containing 12-mer primer, Lane 2 and 5 included fully-extended products, and Lanes 3 and 6 included cleaved FAM-labeled primer after UDG and piperidine treatment. All reactions were carried out at 37 °C for 4 h.

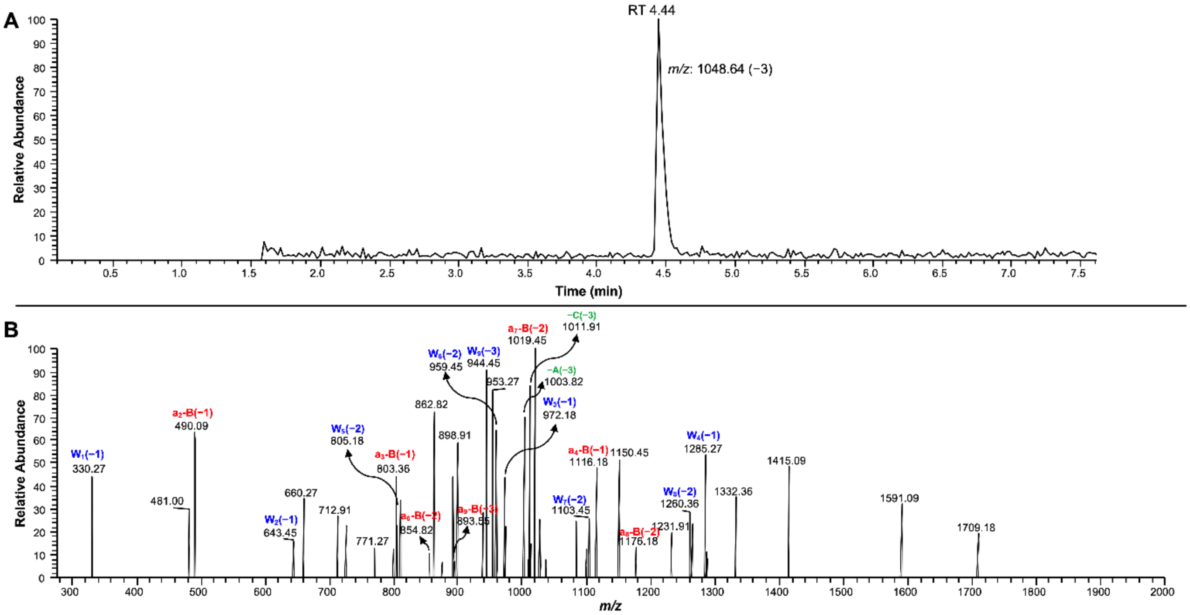

Figure S13. LC-ESI-MS/MS sequencing analysis of full-length extension reactions for control DNA template by hPol η in the presence of dNTPs. *A*, extracted ion chromatogram for *m/z* 1048.64 (−3, t_R 4.44 min); *B*, CID spectrum of *m/z* 1048.64 (−3). See Table S4 for fragment assignment. a-B fragments represented in red, W fragments in blue, and base losses in green.

Table S4

Observed and theoretical CID fragments of *m/z* 1048.64 (−3) from full-length extended products for control DNA template

The extended product sequence is 5′-pAAACTGAGAA-3′ (Figure S13*B*), indicating insertion of T with blunt end addition of A.

Fragment assignment	*m/z* observed	*m/z* theoretical
5′-pAA (a_2-B, −1)	490.09	490.28
5′-pAAA (a_3-B, −1)	803.36	803.49
5′-pAAAC (a_4-B, −1)	1116.18	1116.70
5′-pAAACTG (a_6-B, −2)	854.82	854.54
5′-pAAACTGA (a_7-B, −2)	1019.45	1019.14
5′-pAAACTGAG (a_8-B, −2)	1176.18	1175.75
5′-pAAACTGAGA (a_9-B, −3)	893.55	893.23
pAACTGAGAA-3′ (W_9, −3)	944.45	944.28
pACTGAGAA-3′ (W_8, −2)	1260.36	1260.32
pCTGAGAA-3′ (W_7, −2)	1103.45	1103.71
pTGAGAA-3′ (W_6, −2)	959.45	959.12
pGAGAA-3′ (W_5, −2)	805.18	807.02
pAGAA-3′ (W_4, −1)	1285.27	1285.84
pGAA-3′ (W_3, −1)	972.18	972.63
pAA-3′ (W_2, −1)	643.45	643.42
pA-3′ (W_1, −1)	330.27	330.21

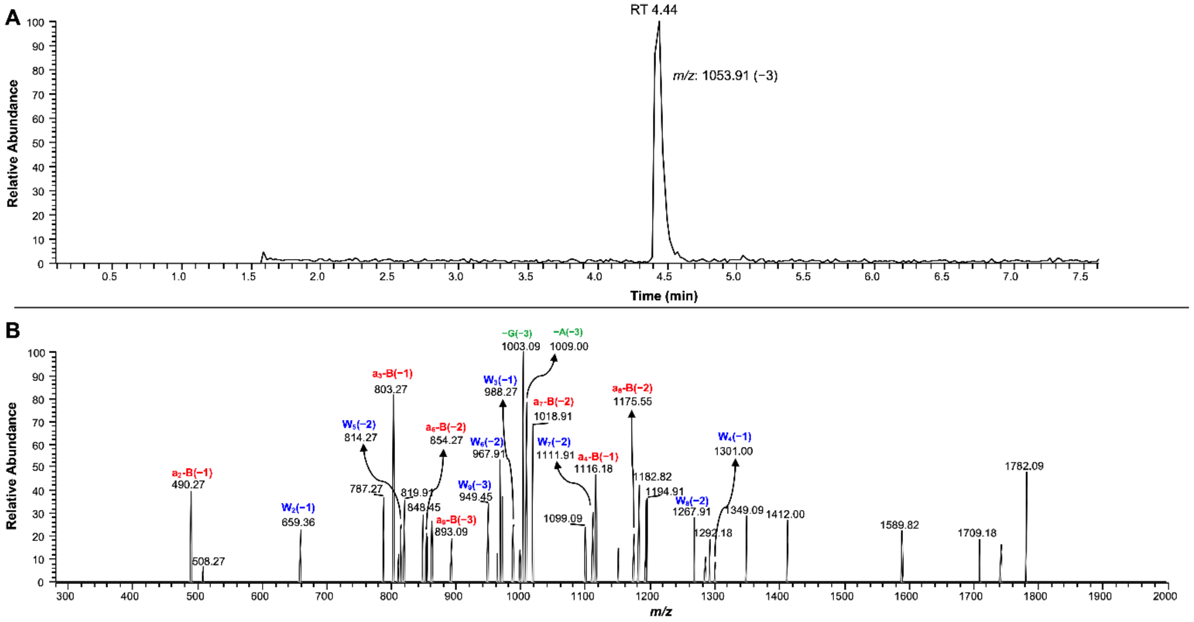

Figure S14. LC-ESI-MS/MS sequencing analysis of full-length extension reactions for control DNA template by hpol η in the presence of dNTPs. *A*, extracted ion chromatogram for *m/z* 1053.91 (−3, t_R 4.44 min); *B*, CID spectrum of *m/z* 1053.91 (−3). See Table S5 for fragment assignments.

Table S5

Observed and theoretical CID fragments of *m/z* 1053.91 (−3) from full-length extended products for control DNA template

The extended product sequence is 5′-pAAACTGAGAG-3′ (Figure S14B), indicating insertion of T with blunt end addition of G.

**Fragment assignment**	***m/z* observed**	***m/z* theoretical**
5′-pAA (a_2-B, −1)	490.27	490.28
5′-pAAA (a_3-B, −1)	803.27	803.49
5′-pAAAC (a_4-B, −1)	1116.18	1116.70
5′-pAAACTG (a_6-B, −2)	854.27	854.54
5′-pAAACTGA (a_7-B, −2)	1018.91	1019.14
5′-pAAACTGAG (a_8-B, −2)	1175.55	1175.75
5′-pAAACTGAGA (a_9-B, −3)	893.09	893.23
pAACTGAGAG-3′ (W_9, −3)	949.45	949.61
pACTGAGAG-3′ (W_8, −2)	1267.91	1268.32
pCTGAGAG-3′ (W_7, −2)	1111.91	1111.71
pTGAGAG-3′ (W_6, −2)	967.91	967.12
pGAGAG-3′ (W_5, −2)	814.27	815.02
pAGAG-3′ (W_4, −1)	1301.00	1301.84
pGAG-3′ (W_3, −1)	988.27	988.63
pAG-3′ (W_2, −1)	659.36	659.42

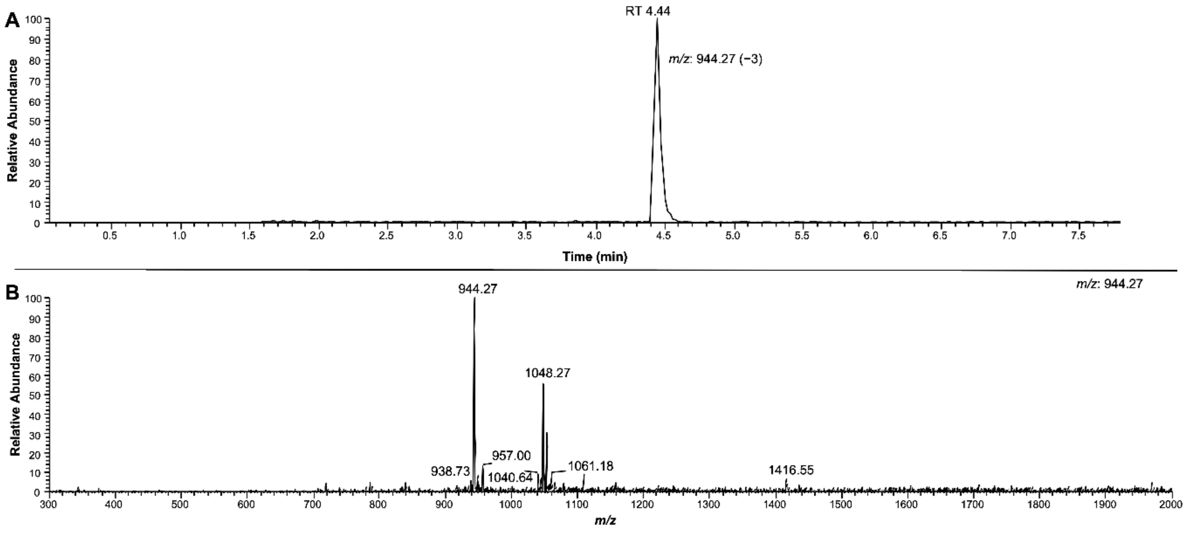

Figure S15. LC-ESI-MS/MS sequencing analysis of full-length extension reactions for peptide-DNA crosslink by hPol η in the presence of dNTPs. *A*, extracted ion chromatogram for *m/z* 944.27 (−3, t_R 4.44 min); *B*, another observed peak *m/z* 1048.68 (−3) eluting at the same t_R. See Table S6 for fragment assignments.

Table S6

Observed and theoretical CID fragments of *m/z* 944.27 (−3) from full-length extended products for peptide-DNA crosslink

The extended product sequence is 5′-pAAACTGAGA-3′ (Fig. 7), indicating the insertion of T.

Fragment assignment	*m/z* observed	*m/z* theoretical
5′-pAA (a_2-B, −1)	490.00	490.28
5′-pAAA (a_3-B, −1)	803.18	803.49
5′-pAAAC (a_4-B, −1)	1116.27	1116.70
5′-pAAACTG (a_6-B, −2)	854.18	854.54
5′-pAAACTGA (a_7-B, −2)	1019.36	1019.14
5′-pAAACTGAG (a_8-B, −2)	1175.45	1175.75
pAACTGAGA-3′ (W_8, −3)	840.09	839.87
pACTGAGA-3′ (W_7, −2)	1103.36	1103.71
pTGAGA-3′ (W_5, −2)	802.27	802.51
pGAGA-3′ (W_4, −2)	650.09	650.41
pAGA-3′ (W_3, −1)	972.45	972.63
pGA-3′ (W_2, −1)	659.18	659.42
pA-3′ (W_1, −1)	330.09	330.21

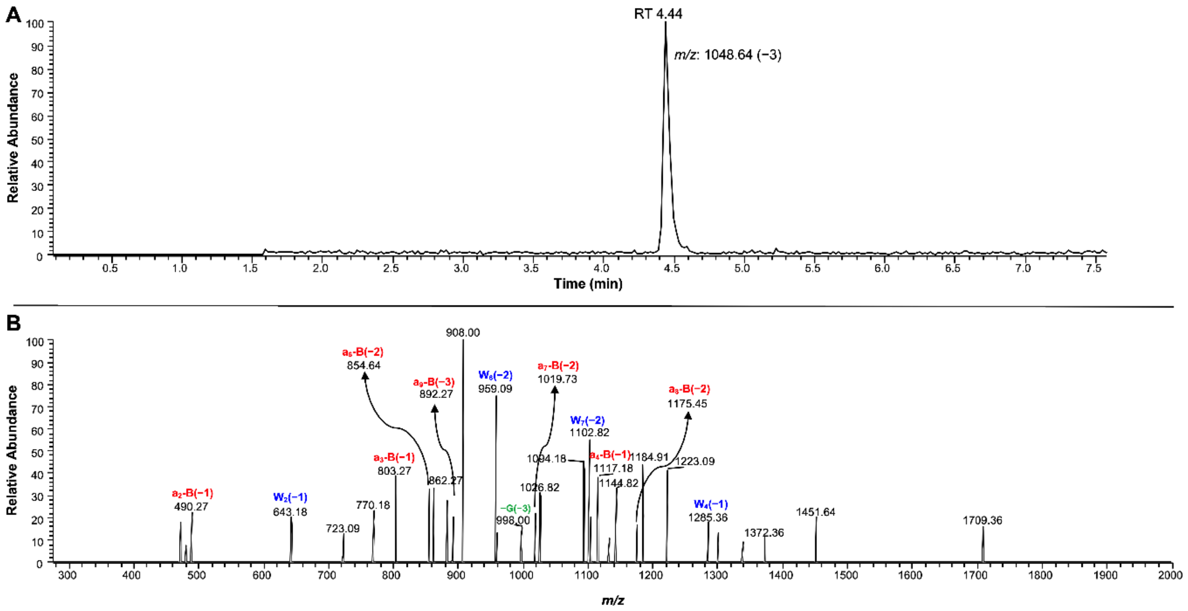

Figure S16. LC-ESI-MS/MS sequencing analysis of full-length extension reactions for peptide-DNA crosslink by hPol η in the presence of dNTPs. *A*, extracted ion chromatogram for *m/z* 1048.64 (−3, t_R 4.44 min); *B*, CID spectrum of *m/z* 1048.64 (−3). See Table S7 for fragment assignments.

Table S7

Observed and theoretical CID fragments of *m/z* 1048.64 (−3) from full-length extended products for peptide-DNA crosslink

The extended product sequence is 5′-pAAACTGAGAA-3′ (Figure S16B), indicating insertion of T with blunt end addition of A.

Fragment assignment	*m/z* observed	*m/z* theoretical
5′-pAA (a_2-B, −1)	490.27	490.28
5′-pAAA (a_3-B, −1)	803.27	803.49
5′-pAAAC (a_4-B, −1)	1117.18	1116.70
5′-pAAACTG (a_6-B, −2)	854.64	854.54
5′-pAAACTGA (a_7-B, −2)	1019.73	1019.14
5′-pAAACTGAG (a_8-B, −2)	1175.45	1175.75
5′-pAAACTGAGA (a_9-B, −3)	892.27	893.23
pCTGAGAA-3′ (W_7, −2)	1102.82	1103.71
pTGAGAA-3′ (W_6, −2)	959.09	959.12
pAGAA-3′ (W_4, −1)	1285.36	1285.84
pAA-3′ (W_2, −1)	643.18	643.42

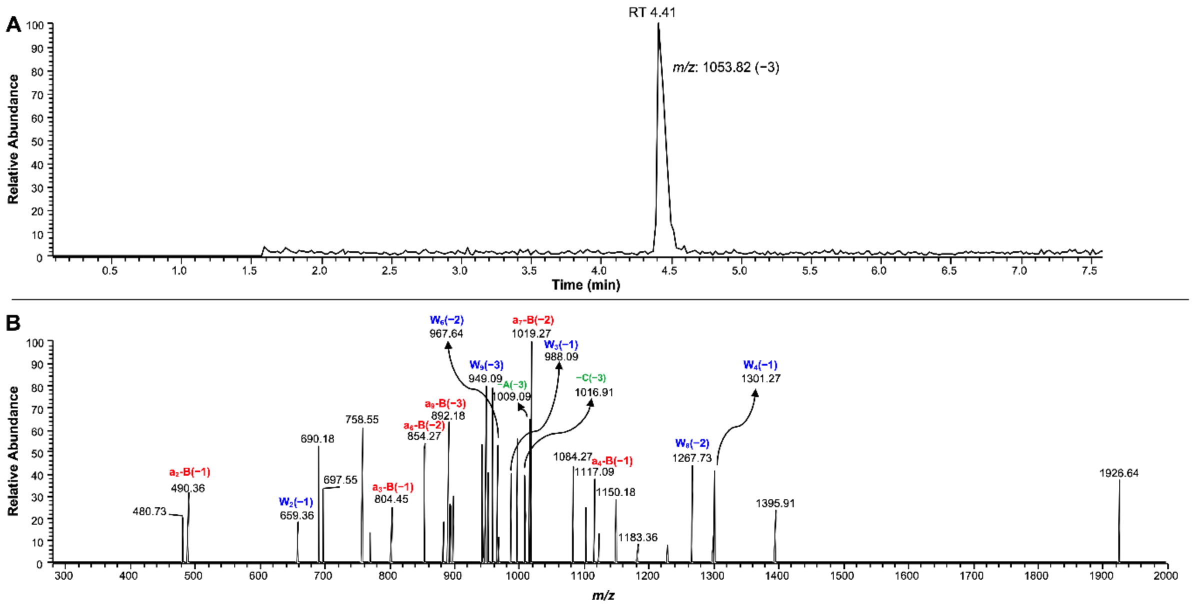

Figure S17. LC-ESI-MS/MS sequencing analysis of full-length extension reactions for peptide-DNA crosslink by hPol η in the presence of dNTPs. *A*, extracted ion chromatogram for *m/z* 1053.82 (−3, t_R 4.41 min); *B*, CID spectrum of *m/z* 1053.82 (−3). See Table S8 for fragment assignment.

Table S8

Observed and theoretical CID fragments of *m/z* 1053.82 (−3) from full-length extended products for peptide-DNA crosslink

The extended product sequence is 5′-pAAACTGAGAG-3′ (Figure S17B), indicating insertion of T with blunt end addition of G.

Fragment assignment	*m/z* observed	*m/z* theoretical
5′-pAA (a_2-B, −1)	490.36	490.28
5′-pAAA (a_3-B, −1)	804.45	803.49
5′-pAAAC (a_4-B, −1)	1117.09	1116.70
5′-pAAACTG (a_6-B, −2)	854.27	854.54
5′-pAAACTGA (a_7-B, −2)	1019.27	1019.14
5′-pAAACTGAGA (a_9-B, −3)	892.18	893.23
pAACTGAGAG-3′ (W_9, −3)	949.09	949.61
pACTGAGAG-3′ (W_8, −2)	1267.73	1268.32
pTGAGAG-3′ (W_6, −2)	967.64	967.12
pAGAG-3′ (W_4, −1)	1301.27	1301.84
pGAG-3′ (W_3, −1)	988.09	988.63
pAG-3′ (W_2, −1)	659.36	659.42
